# Entrapment in the military context: Factor structure and associations with suicidal thoughts and behaviors

**DOI:** 10.1111/sltb.13105

**Published:** 2024-08-13

**Authors:** Justin C. Baker, Sam Cacace, Robert J. Cramer, Susan Rasmussen, Christiana Martin, Alexis M. May, Cynthia Thomsen, AnnaBelle O. Bryan, Craig J. Bryan

**Affiliations:** ^1^ The Ohio State University Columbus Ohio USA; ^2^ University of North Carolina Charlotte North Carolina USA; ^3^ University of Strathclyde Glasgow Scotland; ^4^ Wesleyan University Middletown Connecticut USA; ^5^ Naval Health Research Center San Diego California USA

**Keywords:** entrapment, factor analysis, military, social support, suicide

## Abstract

**Background:**

Improved understanding of how US service members transition from chronic/baseline to acute suicide risk is warranted. One such model, the Integrated Motivational Volitional Model of Suicide, posits entrapment as central to this process. However, entrapment has not been extensively investigated within military populations.

**Methods:**

This study examines the factor structure, reliability, and predictive validity of the Entrapment Scale (E‐Scale) within a military population. Exploratory structural equation modeling (SEM) and confirmatory factor analysis compared one‐ versus two‐factor structures of the E‐Scale. Autoregressive SEM assessed if E‐Scale scores predicted suicidal ideation and suicide attempt likelihood at 6‐ and 12‐month follow‐up, and examined whether the impact of entrapment was moderated by social support (i.e., appraisal, tangible, and belonging).

**Results:**

Results favored a two‐factor solution (external and internal) of entrapment. The relationship between entrapment and suicide outcomes was moderated by perceived social support but in unexpected directions. Unexpectedly, social support strengthened the relationship between external entrapment and suicide outcomes for most models. Only tangible support moderated the relationship between internal entrapment (IE) and suicide outcomes as predicted.

**Conclusions:**

IE is linked with suicidal ideation in the short‐term, whereas external entrapments relationship with suicide outcomes may reflect more persistent social challenges for military members.

## INTRODUCTION

Suicide is a pressing public health concern impacting United States (US) military service members and veteran populations. As suicide rates increased in the past 20 years, military suicide rates have outpaced US civilian rates, with service member deaths at 28.7 suicide deaths per 100,000 and veteran deaths at 31.7 per 100,000 (Department of Defense, 2020; U.S. Department of Veterans Affairs, 2022). Service member suicide deaths also have a substantial effect on military family members (Peterson et al., 2022), who often experience increased shame, stigma, and lack of social support following their loved one's death.

Extensive research focused on identifying preexisting vulnerabilities that place individuals at a greater risk for suicide has expanded our knowledge of risk factors for suicide, including sociodemographic factors, military characteristics, psychosocial factors, and psychiatric symptoms (Nichter et al., 2022). However, despite an improved understanding of individual vulnerabilities that increase risk for suicidal thoughts and behaviors (STBs), the quest for accurate prediction of who will die by suicide continue to fall short (Franklin et al., 2017). The ability to predict when someone at risk for suicide will transition into an acutely suicidal state also remains elusive, hindering efforts to intervene and prevent suicide. Recent models of suicide therefore attempt to both identify vulnerabilities that increase suicide risk and explain the dynamic transition from chronic/baseline risk to acute risk and active suicidal behaviors (Bryan & Rudd, 2018; O'Connor & Kirtley, 2018).

One construct proposed as central to the pathway to acute suicide risk is entrapment, defined as feeling an urgent need to escape aversive conditions that are perceived as uncontrollable, unbearable, and inescapable (Gilbert & Allan, 1998; Taylor et al., 2011). Entrapment is a central component of the Integrated Motivational‐Volitional (IMV) model of Suicide (O'Connor, 2011; O'Connor & Kirtley, 2018). In the following sections we review entrapment as a clinically and theoretically relevant factor in suicide prevention to establish the gaps concerning and benefits of examining it in a military context.

### Entrapment within the context of the integrated motivational volitional model of suicide

The IMV model of suicide adopts an “ideation‐to‐action” (Klonsky & May, 2014) approach to suicide, and identifies entrapment as being central to the transition from defeat to suicidal ideation. The IMV model theorizes that it is the experience of feeling defeated (i.e., failed social struggle triggered by stressful life events or other environmental precipitants) and not being able to escape from this experience (i.e., being trapped) which increases the likelihood of suicidal ideation. While entrapment is core to the IMV, the concept itself is not new. Entrapment as a construct first emerged from the animal literature (Dixon, 1998; Price & Sloman, 1987) and was later incorporated into the arrested flight approach to depression (e.g., Gilbert & Allan, 1998), before also being applied with the suicidology literature (e.g., O'Connor, 2003; Rasmussen et al., 2010; Williams, 2001). Entrapment is typically measured with the Entrapment Scale (E‐Scale; Gilbert & Allan, 1998) which captures a sense of entrapment associated with external life events or circumstances (external entrapment^i^) as well as internal cognitive processes (internal entrapment [IE]^i^). Although the original development of entrapment within the IMV model recognizes two subtypes (i.e., external vs. internal), the proposed IMV pathway (O'Connor & Kirtley, 2018) does not distinguish between these subclasses.

Research examining the IMV model has shown robust evidence for the association between overall entrapment and suicidal ideation (see O'Connor & Portzky, 2018). However, recent research has highlighted the benefits of separately considering the internal and external subdimensions of entrapment both cross‐sectionally (e.g., Carvalho et al., 2013; Cramer et al., 2019; Forkmann et al., 2018) and prospectively (e.g., Höller et al., 2022; Owen et al., 2018). Results from these studies have consistently shown (a) greater support for a two‐factor than a one‐factor model of the E‐Scale, and (b) a greater role for internal than external entrapment in the prediction of suicidal ideation; however, further prospective data across diverse samples is required to allow for a better understanding of the causal pathways to suicidal ideation and behavior (Franklin et al., 2017).

A better understanding of the potential differential impact of internal versus external entrapment may also have clinical implications and could suggest that psychotherapeutic interventions might be better focussed on internal than external entrapment. Initial evidence suggests that internal, but not external entrapment, is an important construct involved in facilitating therapeutic change during crisis‐focused psychotherapy (Tzur Bitan et al., 2019).

### The role of moderators in the integrated motivational volitional model of suicide

A critically understudied component of the IMV model is the role of moderators that can either exacerbate or mitigate the strength of associations in the defeat‐entrapment‐suicidal ideation path (O'Connor & Portzky, 2018). When these factors are present, they allow an individual who is otherwise feeling trapped, to see alternative solutions and options, which may lead to less psychological pain and a more positive outlook (O'Connor & Kirtley, 2018). Conversely, when absent, the theory proposes that there is increased likelihood that entrapment will lead to suicidal ideation.

Within a military setting, there may be value in examining social support (Bryan, 2011; Bryan et al., 2012; Thoresen & Mehlum, 2008) as a potential motivational moderator due to the unique experiences faced by service members, retirees, and their family members. While taking part in military service can provide a strong sense of social support (e.g., Selby et al., 2010), it can also be associated with feelings of low support, difficulty reintegrating into civilian life, and feeling burdensome to family members (Anestis et al., 2009; Brenner et al., 2008; Bryan, 2011; Lusk et al., 2015). Military family members may also experience social isolation and a low sense of support (Strong & Lee, 2017). The way that veterans perceive their social support has been found to be an important factor in the development of suicidal ideation (Lemaire & Graham, 2011; Levi‐Belz et al., 2022; Pietrzak et al., 2010; Shelef et al., 2016). Additionally, social support in military personnel has been differentially linked to suicidal ideation (Bryan & Hernandez, 2013), with emotional distress being associated with increased suicidal ideation at low levels of tangible support (i.e., provision of resources). However, in the same study belonging support (i.e., companionship support) and appraisal support (i.e., provision of advice) were not associated with suicidal ideation. It is important to extend existing work to understand how different types of perceived social support (i.e., appraisal, tangible, and belonging) impact the link between entrapment and STBs in a military sample; however, to date this has not been done within the context of the IMV.

### The present study

Despite evidence that entrapment is associated with elevated risk for suicide, there is limited empirical evidence regarding the association between entrapment and suicide in military‐affiliated populations (Shelef et al., 2016); with only one study with shared coauthors from this study demonstrating that among a military sample IE mediated the relationship among those with positive posttraumatic stress disorder screens and past‐month suicidal ideation and planning as moderated by fearlessness of death (Oakey‐Frost et al., 2022). Given this demonstrated link among symptoms of PTSD and entrapment, exposure to trauma was included as a covariate in analyzed models. However, no studies investigating entrapment within a military context have examined the factor structure of the E‐Scale or the predictive validity of entrapment on STBs. A factor analysis of the E‐Scale is a first step in assessing the cultural sensitivity of entrapment as conceptualized within the IMV model within a military population (O'Connor & Portzky, 2018).

Entrapment may be an especially important psychological risk factor among military personnel owing to institutional practices and cultural norms that restrict service member's autonomy in many areas of life including job duties, re‐assignments, training opportunities, and deployments (Suicide Prevention and Response Independent Review Committee, 2023). The present investigation examined the factor structure, reliability, and predictive validity of entrapment, as assessed by the E‐Scale, among those eligible to receive care within the military healthcare system (i.e., active duty military service members, dependents of service members, and military retirees). Doing so will inform the measurement of entrapment, theoretical application of the IMV model, and clinical utility of social support among military persons seeking medical care. We tested the following hypotheses (H):

H1: A two‐factor entrapment structure will demonstrate better fit to the data than a single‐factor structure.

H2: Internal, but not external entrapment will predict increased suicidal ideation and suicide attempt likelihood at 6‐ and 12‐month follow‐ups.

H3: Perceived social support will moderate the relationship between entrapment scores and suicide outcomes at 6‐ and 12‐ month follow‐ups. More specifically, entrapment should be a stronger predictor of the likelihood of suicidal ideation/attempt likelihood when social support is low versus high.

Given the debate on the importance of covarying depression in studies investigating suicidal ideation (Rogers et al., 2018) and the close link between the arrested flight model, entrapment, and depression (e.g., Gilbert & Allan, 1998) models with and without baseline depression scores are included and discussed.

## METHODS

This study is a secondary analysis of data gathered as part of the PRImary Care Screening Methods (PRISM) study, a multisite prospective cohort study examining universal suicide risk screening efforts within US military primary care clinics (Bryan et al., 2021). A complete description of the rationale and methods of the PRISM study have been previously published, with a brief description of the procedures pertinent to this study included below (Bryan et al., 2019). Institutional Review Board approval of the study's procedures was obtained through the Naval Health Research Center prior to participant enrollment.

### Participants

Participants included 2744 primary care patients ranging in age from 18 to 89 years old (*M* = 40.4, *SD* = 19.6). Full sample demographics and baseline report of lifetime SI and SA are summarized in Table 1. Information regarding missing data and those lost to follow‐up has been previously published and can be found elsewhere (Bryan et al., 2021). Inclusion criteria included being: (1) 18 years of age or older, (2) eligible to receive medical services within the military healthcare system (i.e., active military service members, dependents of active military service members, or military retirees), (3) able to understand and read the English language, and (4) able to complete the informed consent process. Exclusionary criteria were kept purposely minimal to maintain generalizability of results and consisted of having a medical or psychiatric condition that diminished capacity for providing informed consent (e.g., acute intoxication, psychosis).

**TABLE 1:** Participant demographics (*N* = 2744).

Variable	*n* (%)
Gender	
Male	1380 (51.3)
Female	1279 (47.5)
Other	9 (0.3)
Prefer not to answer	17 (0.6)
Unknown / Missing	59 (2.2)
Race	
White/Caucasian	1811 (67.3)
Black/African American	506 (18.8)
Asian	115 (4.3)
Native Amer. Alaska Native	123 (4.6)
Pac. Isl./Native Hawaiian	44 (1.6)
Other	272 (10.1)
Hispanic/Latino Ethnicity	
Yes	415 (15.4)
No	2199 (81.7)
Other	20 (0.7)
Prefer not to answer	51 (1.9)
Military service	
Yes, current member	1652 (61.4)
Yes, in the past	451 (16.8)
No	580 (21.6)
Branch of service	
Air Force	236 (11.2)
Army	408 (19.4)
Coast guard	3 (0.1)
Marines	283 (13.5)
Navy	1171 (55.7)
Prior suicide ideation	
Yes	773 (28.8)
No	1838 (68.5)
Missing	74 (2.8)
Prior suicidal behavior	
Yes	237 (8.8)
No	2356 (87.7)
Missing	92 (3.4)
	M (SD)
Age (years)	40.4 (19.6)

### Procedures

Participants were recruited from six primary care clinics located at five separate military installations from July 2015 to August 2018. The primary care clinics ranged from small community clinics to large medical centers, with participants representing all branches of the US military. The research associate informed interested prospective participants about the study then reviewed and completed the informed consent process. Participants were provided with a Wi‐Fi‐enabled tablet and instructed to complete several electronic surveys as part of the baseline assessment. Follow‐up assessments were completed at 6‐ and 12‐months over the phone by a trained evaluator who assessed for the occurrence of STBs since baseline. At the completion of each phone interview, participants were compensated with a $50 electronic gift card.

### Measures

#### Demographic questionnaire

The demographic questionnaire asked participants to provide their age, gender, race and ethnicity, sexual orientation, military status, and branch of service.

#### E‐Scale

The E‐Scale is a self‐report measure that assesses for subjective experiences of external and internal feelings of being trapped (Gilbert & Allan, 1998). External entrapment (EE) refers to circumstantial factors that contribute to feeling like escape is not possible (e.g., “I can see no way out of my current situation”). IE refers to feeling trapped by unwanted thoughts and feelings (e.g., “I feel trapped inside myself”). The E‐Scale assesses both constructs via a 16‐item measure that asks participants to rate the extent to which each statement represents their current view of themselves (0 = *not at all like me*, to 4 = *extremely like me*); higher scores indicate higher levels of entrapment. Item responses are summed to compute scale scores. Scores for the full‐scale scores range from 0 to 64; EE subscale scores (items 1–10) range from 0 to 40, and IE subscale scores (items 11–16) range from 0 to 24. Across samples, internal (*α* = 0.86–0.95) and external (*α* = 0.89–0.93) subscales of the E‐Scale are rated as having acceptable to good psychometric properties (Cramer et al., 2019; Gilbert & Allan, 1998). In the current sample, the reliability coefficient ω is calculated after confirmatory factor analysis (CFA) establishing the measurement model to satisfy H1.

#### Patient health questionnaire‐8 (PHQ‐8)

The PHQ‐8 (Kroenke et al., 2001) is a widely used 8‐item measure of depression that asks participants to rate the frequency of symptoms that align with symptoms of major depressive disorder as defined by Diagnostic and Statistical Manual of Mental Disorders, 5th Edition (American Psychiatric Association, 2013). PHQ‐8 was used in lieu of PHQ‐9 due to concerns related to potential content contamination of the suicide question (item 9) with the other measures assessing suicide. Symptoms are rated on a 4‐point scale (0 = *not at all*, to 3 = *nearly every day*). Item responses are summed, with higher scores indicating higher depression severity. Estimated reliability coefficients for this study were α = 0.90.

#### Life events checklist (LEC)

Exposure to lifetime traumatic events was measured with the LEC (Gray et al., 2004). The LEC is made up of 16 items assessing potentially traumatic events with a seventeenth item asking about exposure to a type of stressful event other than those listed. For each type of trauma, participants indicated how they had experienced the event: *happened to me, witnessed it, learned about it, part of my job, not sure, or doesn't apply*. Participants can select multiple types of exposure per event. A total score was computed as the number of traumatic events that each participant had. The LEC has demonstrated good test–retest reliability, with the strongest reliability for “*happened to me*” exposure type (Pugach et al., 2021). LEC scores demonstrate strong convergence with measures assessing posttraumatic stress disorder symptoms (Gray et al., 2004). In the current sample, the LEC demonstrated good estimated internal consistency (*KR20* = 0.77).

#### Interpersonal support evaluation list‐shortened version (ISEL‐SV)

The ISEL‐SV is a 12‐item measure of perceived social support (Cohen et al., 1985). Participants were presented with a list of statements and asked to rate whether each statement was true for them (1 = *definitely false*, to 4 = *definitely true*). Item responses are summed with higher scores indicating higher perceptions of social support. There are three subscales consisting of 4‐items each: Appraisal Support (e.g., “I have someone I can turn to for advice”), Belonging Support (e.g., “I have someone I can turn to when I am lonely”), and Tangible Support (e.g., “I have someone I can turn to for practical help”). The ISEL‐SV shows good internal consistency with a Cronbach's alpha score of 0.88–0.90 within a general population (Cohen et al., 1985). Reliability estimates in the present sample were: appraisal support (*α* = 0.68), belonging support (*α* = 0.72), and tangible support (*α* = 0.57).

#### Self‐injurious thoughts and behaviors interview (SITBI)

The SITBI (Nock et al., 2007) is a structured clinician‐administered assessment. In the present study, it was used to assess suicidal ideation (“In the past six months, have you had thoughts of killing yourself?”) and suicide attempt likelihood (“What do you think the likelihood is that you will have thoughts of killing yourself in the future?”). The first item was scored as yes or no; responses on the second item were made on a 5‐point scale (0 = *not at all*, to 4 = *extremely*). The SITBI demonstrates strong interrater reliability and test–retest reliability, as well as strong convergence with other measures of suicidal ideation and suicide attempt (Nock et al., 2007).

### Data analyses

We divided the sample for analyses using a split‐sample technique where three subsamples were randomly generated without replacement. For H1, exploratory structural equation modeling (ESEM) was used to test a one‐versus two‐factor E‐Scale structure in subsample 1. The ESEM approach allows a more conservative model than a typical exploratory factor analysis (Asparouhov & Muthén, 2009), given that the E‐Scale has prior psychometric work in other samples (e.g., Cramer et al., 2019; Gilbert & Allan, 1998). ESEM also allows cross‐loadings between all items but permits restrictions on factor correlations and utilizes variance–covariance matrices to derive model fit (Asparouhov & Muthén, 2009). Continuing H1 analyses, we used CFA to replicate the two‐factor E‐Scale structure in subsample 2. ESEM and CFA were conducted using the fixed‐factor variance approach, where factor variances were set to “1” for identification purposes, and full information maximum‐likelihood estimation (FIML) was used to account for missing data. To obtain reliability coefficients, McDonald's ω (Zinbarg et al., 2006) was calculated in the final CFA two‐factor model for IE and EE. The model was bootstrapped 1000 times to obtain bias‐corrected confidence intervals. Omnibus fit was assessed for each model using suggested cutoff parameters for χ^2^, Comparative Fit Index (CFI), Tucker Lewis Index (TLI), Root Mean Square Error of Approximation (RMSEA), and Standardized Root Mean Square Residual (SRMR), per Hu and Bentler's recommendations (1999).

To test H2 and H3, we conducted three autoregressive SEMs using subsample 3. Each model included: (1) baseline IE and EE; (2) baseline depression and trauma exposure as covariates given their salience in the military population and links to STBs (e.g., Holliday et al., 2020; Ritchie et al., 2003); (3) 6‐ and 12‐month suicidal ideation and suicide attempt likelihood, and; (4) one of three social support subscales (i.e., appraisal support, belonging support, or tangible support). Three additional models were run where depression was excluded as a covariate. The autoregressive effects of baseline suicidal ideation and attempt likelihood were controlled for by creating covariance paths across time points and between baseline ideation and attempt likelihood (Selig & Little, 2012). Maximum Likelihood (ML) estimation with logit links for paths (to account for categorical outcomes) was used for all models. We added one social support subscale per model as a moderator to separate the variance in suicidal ideation and attempt likelihood accounted for by each type of support. Social support subscales were mean‐centered for each model for ease of interpretation.

## RESULTS

### Hypothesis 1

Measures of central tendency for included measures are provided in Table 2. Using the first subsample (*n* = 779), the E‐Scale single‐factor ESEM yielded poor fit to the data (*χ* ^2^(104) = 2102, *p* < 0.001; CFI = 0.83; TLI = 0.80; SRMR = 0.06; RMSEA = 0.16; see Table 3 &4). Further, while all E‐Scale item factor loadings were significant and substantial (λ_range_ = 0.51–0.84, *p*s <0.001), residual variances indicated localized ill‐fit, exceeding 0.1 for all items. The E‐Scale two‐factor solution demonstrated improved fit to the data (*χ* ^2^(89) = 813.08, *p* < 0.001; CFI = 0.94; TLI = 0.0.92; SRMR = 0.03; RMSEA = 0.10; see Table 4), especially in fit indices with parsimony corrections, though *χ* ^2^ remained significant as can be expected with larger samples (Floyd & Wideman, 1995). Further, item factor loadings were significant and substantial (IE λ_range_ = 0.48–0.97, *p*s <0.001; EE λ_range_ = 0.62–0.91, *p*s <0.001) and in the expected direction for their respective factors. Additionally, the correlation between EE and IE was relatively high (*r* = 0.71, *p* < 0.001), indicating shared variance between factors. While residual variances still indicted localized ill‐fit, residuals were lower in the two‐factor solution than in the one‐factor solution. Overall, the E‐Scale two‐factor structure demonstrated meaningful improvement in model fit (Δ*χ* ^2^(1) = 738.85, *p* < 0.0001). Thus, the E‐Scale two‐factor structure was retained in CFA.

**TABLE 2:** Measures of central tendency.

Variable	M	Median	SD	S.E.	Range
PHQ‐8	5.8	4	5.7	0.11	24
E‐Scale	9.0	2	13.8	0.28	64
LEC	3.5	3	3.0	0.06	17
Appraisal	12.8	13	2.9	0.06	12
Belonging	12.3	12	3.0	0.06	12
Tangible	12.4	12	27	0.05	12

Abbreviations: M, mean; SD, standard deviation; S.E, standard error.

**TABLE 3:** E‐scale measurement model.

Model	Factor items	Stand. λ	*S.E*.	*p*
1‐Factor ESEM	Entrapment			
	1	0.80	0.01	< 0.001
	2	0.84	0.01	< 0.001
	3	0.51	0.03	< 0.001
	4	0.83	0.01	< 0.001
	5	0.85	0.01	< 0.001
	6	0.83	0.01	< 0.001
	7	0.83	0.01	< 0.001
	8	0.69	0.02	< 0.001
	9	0.81	0.01	< 0.001
	10	0.80	0.01	< 0.001
	11	0.74	0.02	< 0.001
	12	0.80	0.01	< 0.001
	13	0.76	0.02	< 0.001
	14	0.76	0.02	< 0.001
	15	0.79	0.01	< 0.001
	16	0.84	0.01	< 0.001
2‐Factor ESEM	External Entrapment			
	1	0.83	0.02	< 0.001
	2	0.62	0.03	< 0.001
	3	0.60	0.04	< 0.001
	4	0.63	0.03	< 0.001
	5	0.70	0.03	< 0.001
	6	0.77	0.03	< 0.001
	7	0.83	0.03	< 0.001
	8	0.91	0.03	< 0.001
	9	0.84	0.02	< 0.001
	10	0.90	0.03	< 0.001
	11	0.15	0.04	< 0.001
	12	0.39	0.04	< 0.001
	13	0.00	0.00	0.119
	14	−0.06	0.04	0.081
	15	0.14	0.04	< 0.001
	16	0.39	0.04	< 0.001
	Internal Entrapment			
	1	0.00	0.02	0.856
	2	0.28	0.04	< 0.001
	3	−0.08	0.05	0.117
	4	0.25	0.04	< 0.001
	5	0.20	0.04	< 0.001
	6	0.10	0.04	0.007
	7	0.04	0.04	0.315
	8	−0.20	0.04	< 0.001
	9	0.01	0.03	0.789

	10	−0.06	0.04	0.100
	11	0.68	0.04	< 0.001
	12	0.48	0.04	< 0.001
	13	0.88	0.01	< 0.001
	14	0.97	0.03	< 0.001
	15	0.76	0.03	< 0.001
16	0.54	0.03	< 0.001
2‐Factor CFA	External Entrapment			
	1	0.84	0.01	< 0.001
	2	0.86	0.01	< 0.001
	3	0.57	0.03	< 0.001
	4	0.81	0.01	< 0.001
	5	0.82	0.01	< 0.001
	6	0.84	0.01	< 0.001
	7	0.82	0.01	< 0.001
	8	0.77	0.02	< 0.001
	9	0.83	0.01	< 0.001
	10	0.83	0.01	< 0.001
	Internal Entrapment			
	11	0.85	0.01	< 0.001
	12	0.84	0.01	< 0.001
	13	0.90	0.01	< 0.001
	14	0.92	0.01	< 0.001
	15	0.86	0.01	< 0.001
	16	0.87	0.01	< 0.001

Abbreviations: CFA, confirmatory factor analysis; ESEM, exploratory structural equation model.

**TABLE 4:** Exploratory structural equation model and confirmatory factor analysis E‐scale fit statistics.

Model	χ^2^	*Df*	*p* χ^2^	|Δχ2|	|Δdf|	CFI	|ΔCFI|	TLI	SRMR	RMSEA	95% CI	*p* RMSEA <0.05
1‐Factor ESEM	2102.088	104.000	< 0.001			0.829		0.802	0.064	0.157	0.151 | 0.163	< 0.001
2‐Factor ESEM	813.076	89.000	< 0.001	1289.012	15	0.938	0.109	0.916	0.028	0.102	0.096 | 0.109	< 0.001
2‐Factor CFA	901.025	103.000	< 0.001	87.949	14	0.934	0.004	0.923	0.035	0.100	0.094 | 0.106	< 0.001

Abbreviations: CFA, confirmatory factor analysis; CFI, comparative fit index; df, degrees of freedom; ESEM, exploratory structural equation model; *p*, *p*‐values; RMSEA, root mean squared error of approximation; SRMR, standardized root mean square residual; TLI, tucker‐lewis index; Δ, Change.

Using the second subsample (*n* = 777), the E‐Scale two‐factor CFA restricted cross‐loadings between the EE and IE factors. The E‐Scale two‐factor CFA yielded similar fit to the data as the E‐Scale two‐factor ESEM (*χ* ^2^(103) = 901.03, *p* < 0.001; CFI = 0.93; TLI = 0.92; SRMR = 0.04; RMSEA = 0.10; see Table 2 & 3). Again, *χ* ^2^ remained significant, as can be expected with larger samples (Floyd & Wideman, 1995). Loadings were significant and substantial for all items (IE λ_range_ = 0.57–0.86, *p*s <0.001; EE λ_range_ = 0.62–0.91, *p*s <0.001), and the correlation between EE and IE factors was high (*r* = 0.84, *p* < 0.001), as could be expected with restricted cross‐loadings between items. Residual variances indicated localized ill‐fit, though improved from the single‐factor solution (see Table 3). Reliability coefficients indicated excellent reliability for EE (95% CI ω = 0.93 | 0.95), but marginal reliability for IE (95% CI ω = 0.63 | 0.66). The E‐Scale two factor model was considered adequate for inspection of H2 and H3.

### Hypotheses 2 and 3^ii^

#### Appraisal support model

**FIGURE 1:**
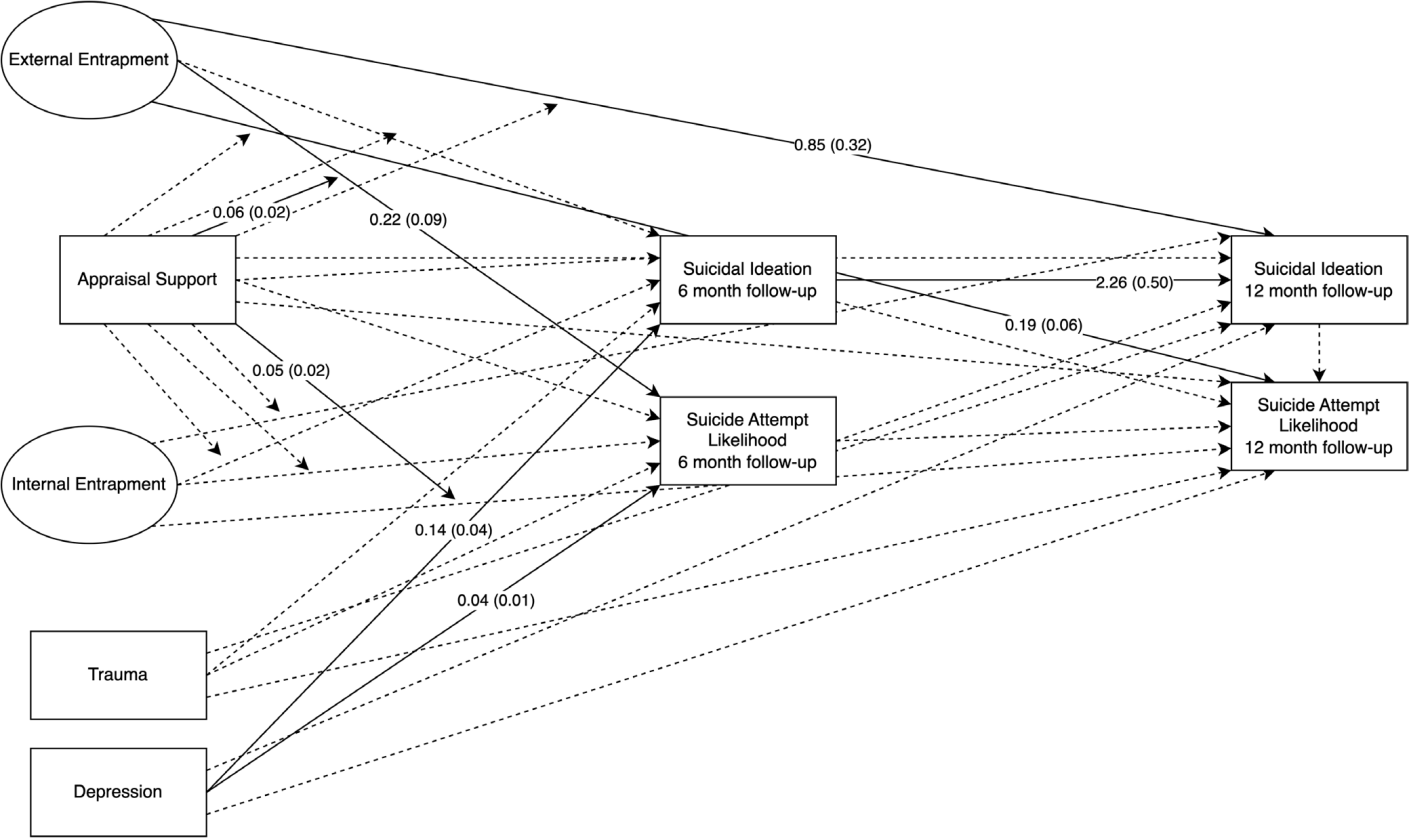
Appraisal support autoregressive model—depression included as covariate. Autoregressive structural equation model results shown with circles representing latent variables (External and Internal entrapment) and rectangles indicating observed variables. Parameter estimates for significant paths shown as unstandardized logistic regression coefficients with standard errors in parentheses. Solid lines indicate significant paths. Dashed lines indicate nonsignificant paths. Arrows pointing directly to variables indicate direct associations between variables, while arrows pointing to paths indicate interactions. Variances and disturbances not shown for brevity.

**FIGURE 2:**
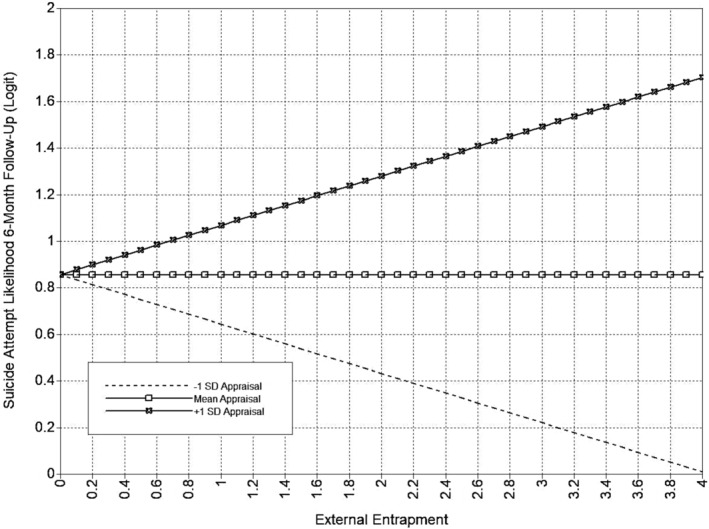
EE × appraisal support interaction (SA 6‐month)—depression included as covariate. Predicted values from interaction of appraisal support with External Entrapment in logit scale of the outcome (suicide attempt likelihood at 6‐month follow‐up). External Entrapment shown at logical values in the metric of the E‐Scale (0–4).

**FIGURE 3:**
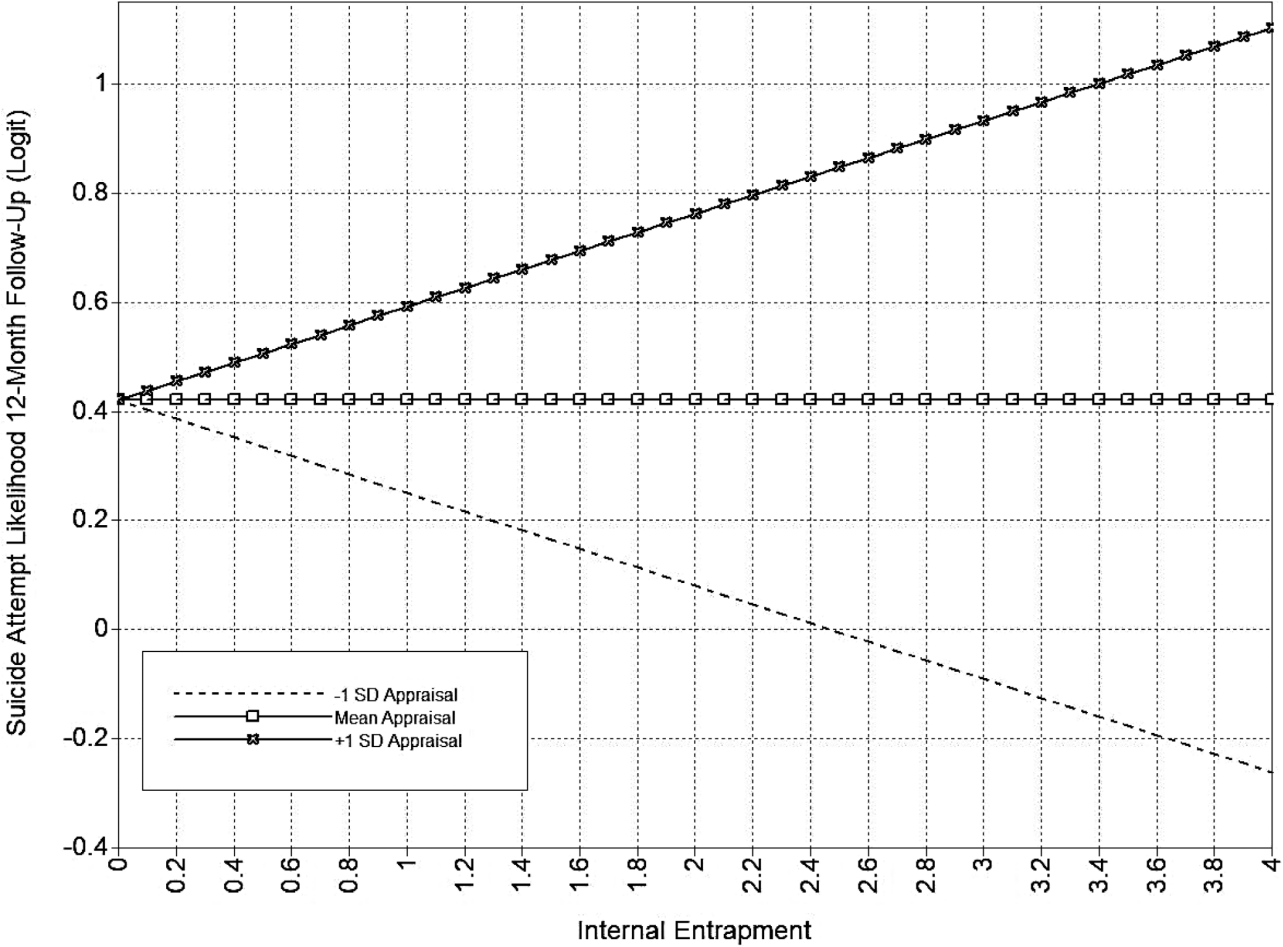
IE × Appraisal Support Interaction (SA 12‐month)—Depression Included as Covariate. Predicted values from interaction of appraisal support with Internal Entrapment in logit scale of the outcome (suicide attempt likelihood at 12‐month follow‐up). Internal Entrapment shown at logical values in the metric of the E‐Scale (0–4).

The full appraisal support model is presented in Table 5 and Figure 1. In the appraisal support model (*LL* = −14,703.19; *AIC* = 29,580.37; *BIC* = 29,986.84), EE, but not IE, had a positive relationship with suicide attempt likelihood at 6‐month follow‐up. Also, the interaction between EE and appraisal support was significant for suicide attempt likelihood at 6‐month follow‐up. Inspection of the interaction using simple slopes showed that the EE‐suicide attempt likelihood was significant across levels of appraisal support and strengthened as appraisal support increases (see Table 6; Figure 2). Additionally, EE, but not IE, had a significant positive relationship with both suicidal ideation and suicide attempt likelihood at 12‐month follow‐up. The interaction between IE and appraisal support was significant for suicide attempt likelihood at 12‐month follow‐up. IE‐suicide attempt likelihood relationship was not significant at low levels of appraisal support, but became significant and positive, strengthening from moderate to high levels of appraisal support (see Table 6; Figure 3).

**TABLE 5:** Entrapment and support type autoregressive model statistics.

Outcome	Predictor	Coef.	*S.E*.	*OR*	*p*	95% CI	Coef.	*S.E*.	*OR*	*p*	95% CI	Coef.	*S.E*.	*OR*	*p*	95% CI
		Appraisal Support					Belonging Support					Tangible Support				
6 mo. SI	EE	0.24	0.24	1.27	0.319	0.79 | 2.03	0.15	0.24	1.17	0.514	0.74 | 1.85	0.11	0.24	1.12	0.638	0.70 | 1.78
	IE	0.35	0.23	1.42	0.135	0.90 | 2.24	0.40	0.23	1.49	0.090	0.94 | 2.35	**0.53**	**0.24**	**1.69**	**0.028**	**1.06 | 2.71**
	EE x Support	0.08	0.07	1.08	0.264	0.95 | 1.23	0.05	0.06	1.05	0.378	0.94 | 1.18	−0.07	0.08	0.93	0.346	0.80 | 1.08
	IE x Support	−0.08	0.06	0.92	0.202	0.82 | 1.04	−0.04	0.06	0.96	0.535	0.86 | 1.08	0.08	0.07	1.08	0.299	0.94 | 1.24
	Trauma Exposure	−0.10	0.06	0.90	0.087	0.81 | 1.02	−0.08	0.06	0.92	0.159	0.82 | 1.03	−0.09	0.06	0.92	0.135	0.82 | 1.03
	Depression	**0.14**	**0.04**	**1.15**	**< 0.001**	**1.07 | 1.23**	**0.13**	**0.03**	**1.14**	**< 0.001**	**1.07 | 1.22**	**0.14**	**0.03**	**1.15**	**< 0.001**	**1.07 | 1.23**
	Support	0.11	0.07	1.12	0.112	0.97 | 1.29	0.04	0.06	1.04	0.486	0.93 | 1.17	**0.15**	**0.08**	**1.17**	**0.041**	**1.01 | 1.35**
6 mo. SA	**EE**	**0.22**	**0.09**		**0.010**		0.16	0.08		0.055		0.15	0.08		0.077	
	IE	0.16	0.09		0.064		0.16	0.09		0.063		**0.21**	**0.09**		**0.018**	
	EE x Support	**0.06**	**0.02**		**0.002**		**0.04**	**0.02**		**0.037**		−0.02	0.03		0.368	
	IE x Support	−0.04	0.02		0.059		−0.04	0.02		0.103		0.05	0.02		0.051	
	Trauma Exposure	−0.02	0.02		0.237		−0.02	0.02		0.258		−0.02	0.02		0.307	
	Depression	**0.04**	**0.01**		**0.001**		**0.03**	**0.01**		**0.002**		**0.03**	**0.01**		**0.002**	
	Support	0.03	0.02		0.117		0.01	0.02		0.584		0.02	0.02		0.300	
12 mo. SI	EE	**0.85**	**0.32**	**2.34**	**0.008**	**1.25 | 4.37**	**0.96**	**0.33**	**2.60**	**0.003**	**1.38 |4.92**	**1.16**	**0.33**	**3.20**	**<0.001**	**1.67 | 6.13**
	IE	−0.11	0.33	0.89	0.734	0.47 | 1.70	−0.35	0.33	0.71	0.295	0.37 | 1.35	−0.65	0.34	0.52	0.057	0.27 | 1.02
	EE x Support	−0.01	0.09	0.99	0.891	0.84 | 1.17	0.06	0.07	1.06	0.450	0.92 | 1.22	**0.35**	**0.13**	**1.42**	**0.008**	**1.09 | 1.84**
	IE x Support	0.09	0.08	1.09	0.283	0.93 | 1.28	−0.01	0.08	0.99	0.882	0.85 | 1.15	**0.31**	**0.12**	**0.73**	**0.010**	**0.58 | 0.93**
	Trauma Exposure	−0.14	0.08	0.87	0.095	0.75 | 1.02	−0.13	0.08	0.88	0.119	0.75 | 1.03	−0.13	0.09	0.88	0.140	0.74 | 1.04
	Depression	0.01	0.05	1.01	0.905	0.92 | 1.10	0.00	0.05	1.00	0.964	0.91 | 1.10	−0.01	0.05	0.99	0.889	0.90 | 1.10
	Support	−0.09	0.08	0.92	0.244	0.79 | 1.06	**−0.16**	**0.07**	**0.85**	**0.014**	**0.75 | 0.97**	**−0.22**	**0.09**	**0.80**	**0.011**	**0.68 | 0.95**
	6 mo. SI	**2.26**	**0.50**	**9.55**	**< 0.001**	**3.61 | 25.24**	**2.25**	**0.54**	**9.48**	**<0.001**	**3.27 | 27.46**	**2.60**	**0.60**	**13.51**	**<0.001**	**4.16 | 43.92**
	6 mo. SA	0.17	0.23	1.18	0.466	0.76 | 1.84	0.21	0.22	1.24	0.342	0.80 | 1.91	0.22	0.24	1.25	0.365	0.77 | 2.01
12 mo. SA	EE	**0.19**	**0.06**		**0.003**		**0.18**	**0.06**		**0.004**		**0.19**	**0.06**		**0.002**	
	IE	−0.02	0.07		0.713		−0.07	0.07		0.288		−0.11	0.07		0.093	
	EE x Support	−0.03	0.02		0.108		−0.02	0.02		0.191		0.04	0.02		0.051	
	IE x Support	**0.05**	**0.02**		**0.019**		0.02	0.02		0.289		**−0.06**	**0.02**		**0.004**	
	Trauma Exposure	0.00	0.01		0.840		0.01	0.01		0.649		0.00	0.01		0.722	
	Depression	−0.01	0.01		0.570		−0.01	0.01		0.485		−0.01	0.01		0.595	
	Support	0.00	0.01		0.749		−0.03	0.01		0.031		−0.02	0.01		0.094	
	6 mo. SI	**1.23**	**0.12**		**< 0.001**		**1.17**	**0.12**		**<0.001**		**0.29**	**0.05**		**<0.001**	
	6 mo. SA	**0.25**	**0.05**		**< 0.001**		**0.28**	**0.05**		**<0.001**		**1.17**	**0.13**		**<0.001**	

*Note*: Bolded terms indicate significance.

Abbreviations: IE, internal entrapment; EE, external entrapment; Support, support type (appraisal, belonging, tangible); x, interaction term; SA model outcomes are continuous while SI model outcomes are binary; +/− 1 SD, One standard deviation above or below the mean; 12 mo; SA, Suicide attempt likelihood at 12 month follow‐up; 12 mo. SI, Suicidal ideation at 12 month follow‐up; 6 mo. SA, Suicide attempt likelihood at 6 month follow‐up; 6 mo. SI, Suicidal ideation at 6 month follow‐up.

**TABLE 6:** Autoregressive model social support simple slopes analyses.

Outcome	Interaction	Level	Coef.	*OR*	*p*	95%CI
6 mo. SA	EE x App					
		‐1*SD*	0.645		< 0.05	0.30 | 0.99
		Mean	0.856		< 0.05	0.55 | 1.16
		+1*SD*	1.068		< 0.05	0.72 | 1.41
12 mo. SA	IE x App					
		‐1*SD*	0.250		*ns*	−0.06 | 0.56
		Mean	0.421		< 0.05	0.15 | 0.69
		+1*SD*	0.591		< 0.05	0.29 | 0.89
6 mo. SA	EE x Belong					
		‐1*SD*	0.50		< 0.05	0.23 | 0.77
		Mean	0.64		< 0.05	0.39 | 0.89
		+1*SD*	0.78		< 0.05	0.48 | 1.08
12 mo. SI	EE x Tang					
		‐1*SD*		0.41	< 0.05	0.20 | 0.63
		Mean		1.07	*ns*	0.74 | 1.40
		+1*SD*		2.76	< 0.05	1.60 | 3.91
12 mo. SI	IE x Tang					
		‐1*SD*		4.83	< 0.05	2.48 | 7.18
		Mean		2.07	*ns*	1.44 | 3.51
		+1*SD*		0.89	*ns*	0.53 | 1.25
12 mo. SA	IE x Tang					
		‐1*SD*	1.87		< 0.05	1.58 | 2.15
		Mean	1.71		< 0.05	1.45 | 1.98
		+1*SD*	1.55		< 0.05	1.27 | 1.84

Abbreviations: App, APpraisal support; Belong, belonging support; CI, confidence interval; EE, external entrapment; IE, internal entrapment; OR, odds ratio; *p*, *p*‐value; Tang, tangible support; x, Interaction term.+/− 1 SD, One standard deviation above or below the mean; 12 mo. SA, Suicide attempt likelihood at 12 month follow‐up; 12 mo. SI, Suicidal ideation at 12 month follow‐up; 6 mo. SA, Suicide attempt likelihood at 6 month follow‐up; 6 mo. SI, Suicidal ideation at 6 month follow‐up.

#### Belonging support model

**FIGURE 4:**
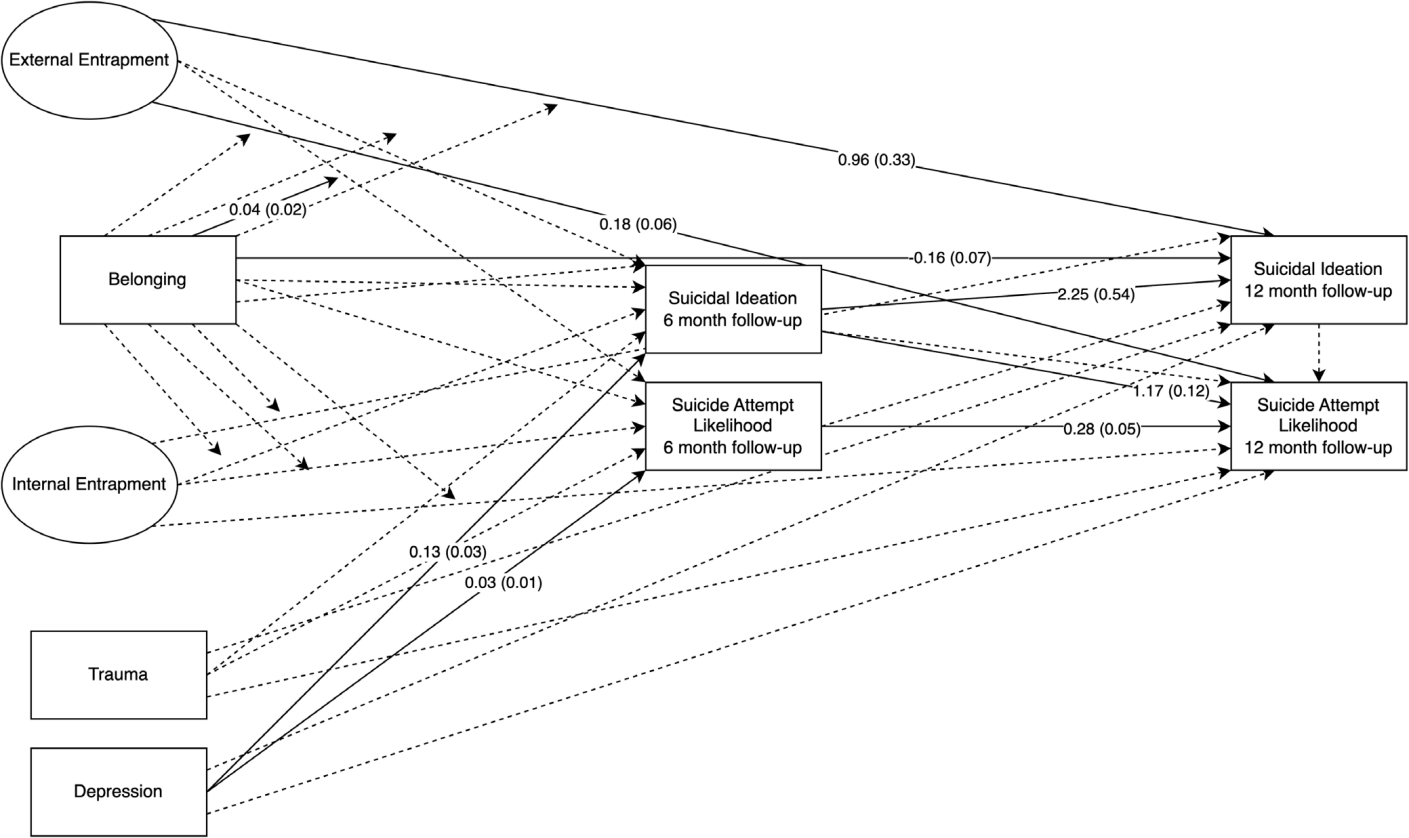
Belonging support autoregressive model—depression included as covariate. Autoregressive structural equation model results shown with circles representing latent variables (External and Internal entrapment) and rectangles indicating observed variables. Parameter estimates for significant paths shown as unstandardized logistic regression coefficients with standard errors in parentheses. Solid lines indicate significant paths. Dashed lines indicate nonsignificant paths. Arrows pointing directly to variables indicate direct associations between variables, while arrows pointing to paths indicate interactions. Variances and disturbances not shown for brevity.

**FIGURE 5:**
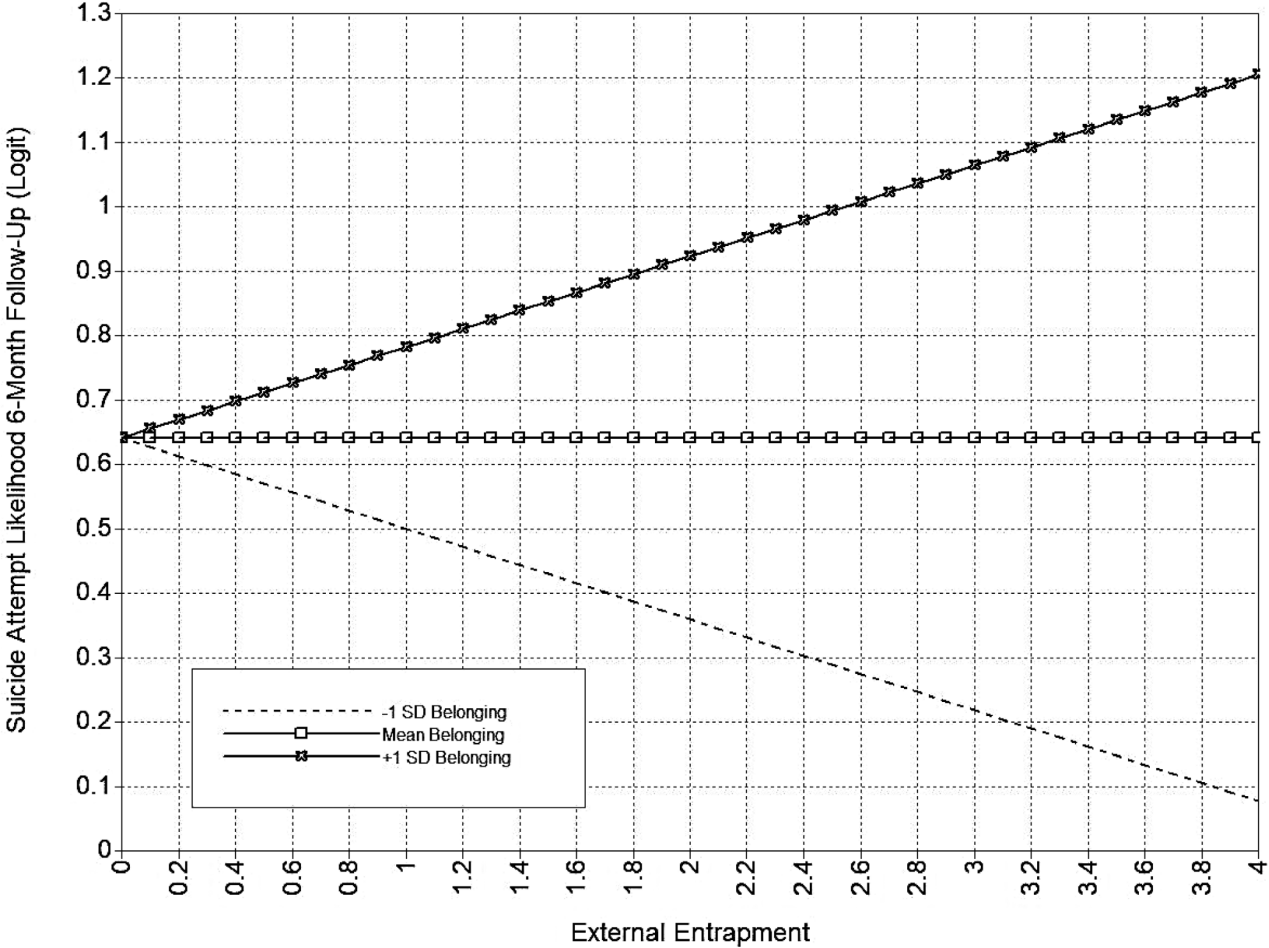
EE × belonging support interaction (SA 6‐month)—depression included as covariate. Predicted values from interaction of belonging support with External Entrapment in logit scale of the outcome (suicide attempt likelihood at 6‐month follow‐up). External Entrapment shown at logical values in the metric of the E‐Scale (0–4).

The full belonging support model is presented in Table 5 and Figure 4. In the belonging support model (*LL* = −14,709.84, *AIC* = 29,593.68; *BIC* = 30,000.15), there were no significant relationships involving EE, IE, or interaction terms with belonging support on suicidal ideation at 6‐month follow‐up. Although, neither EE nor IE had a significant relationship with suicide attempt likelihood at 6‐month follow‐up, the interaction between EE and belonging support was significant for suicide attempt likelihood at 6‐month follow‐up (see Table 6; Figure 5). The EE‐suicide attempt likelihood relationship was significant across levels of belonging support and strengthened as belonging support increased. EE was significantly positively associated with suicidal ideation and suicide attempt likelihood at 12‐month follow‐up. IE nor any entrapment‐belonging support interactions displayed significant associations with suicidal ideation or suicide attempt likelihood at 12‐month follow‐up.

#### Tangible support model

**FIGURE 6:**
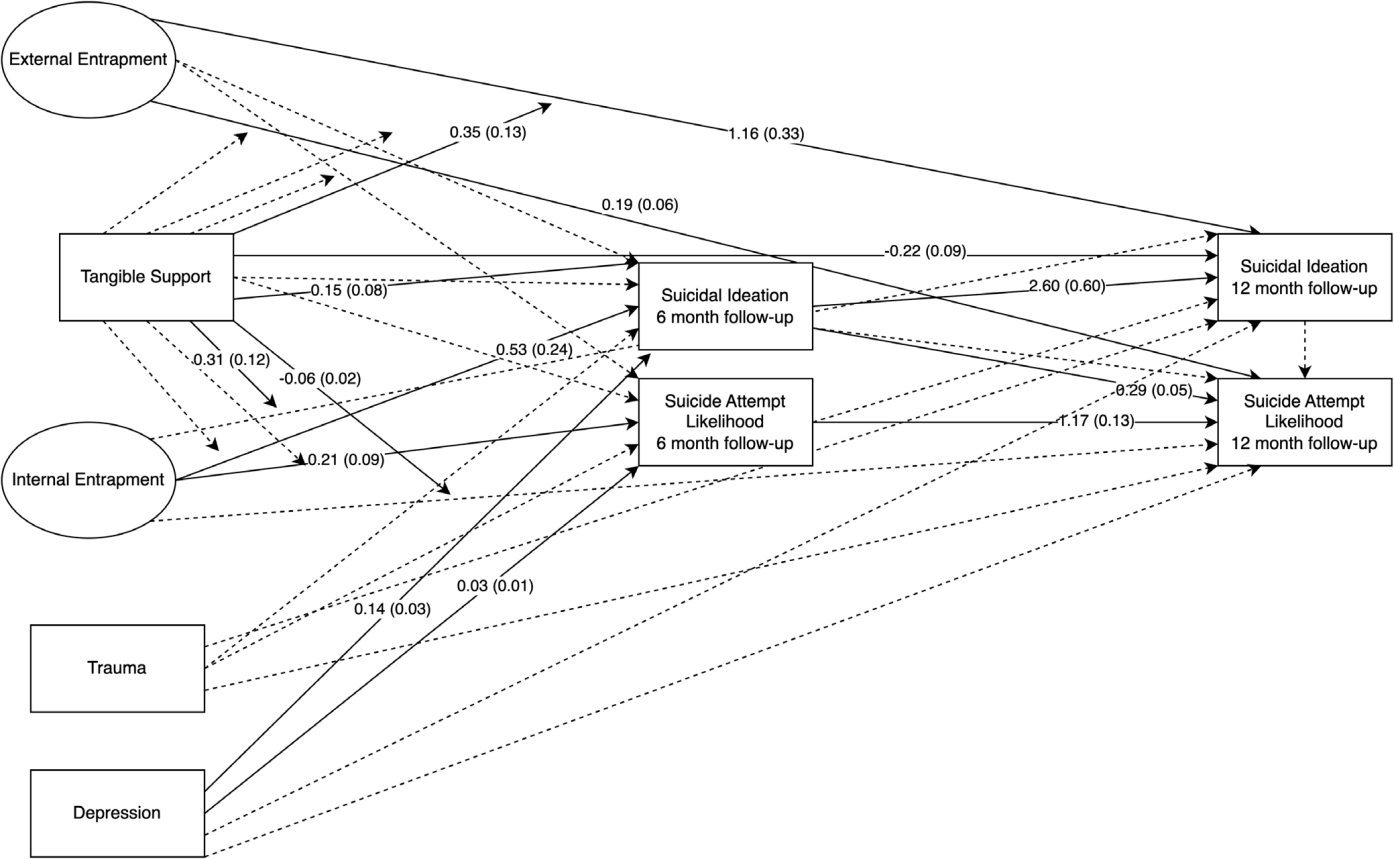
Tangible support autoregressive model—depression included as covariate. Autoregressive structural equation model results shown with circles representing latent variables (External and Internal entrapment) and rectangles indicating observed variables. Parameter estimates for significant paths shown as unstandardized logistic regression coefficients with standard errors in parentheses. Solid lines indicate significant paths. Dashed lines indicate nonsignificant paths. Arrows pointing directly to variables indicate direct associations between variables, while arrows pointing to paths indicate interactions. Variances and disturbances not shown for brevity.

**FIGURE 7:**
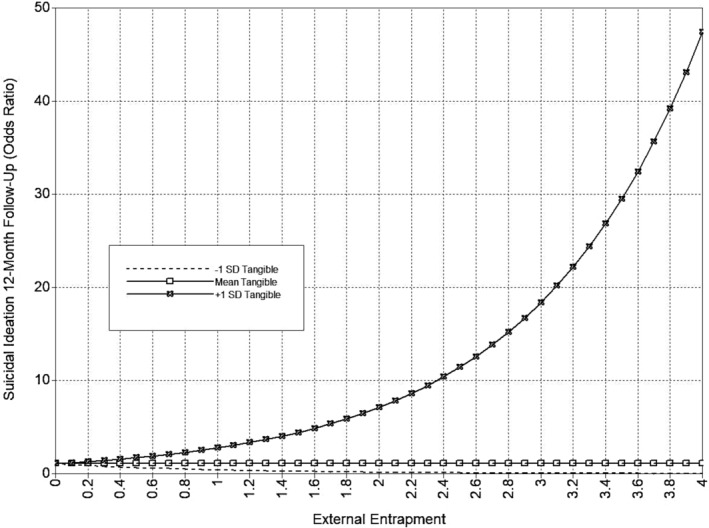
EE × tangible support interaction (SI 12‐month)—depression included as covariate. Predicted values from interaction of tangible support with External Entrapment in odds ratio scale of the outcome (suicidal ideation at 12‐month follow‐up). External Entrapment shown at logical values in the metric of the E‐Scale (0–4).

**FIGURE 8:**
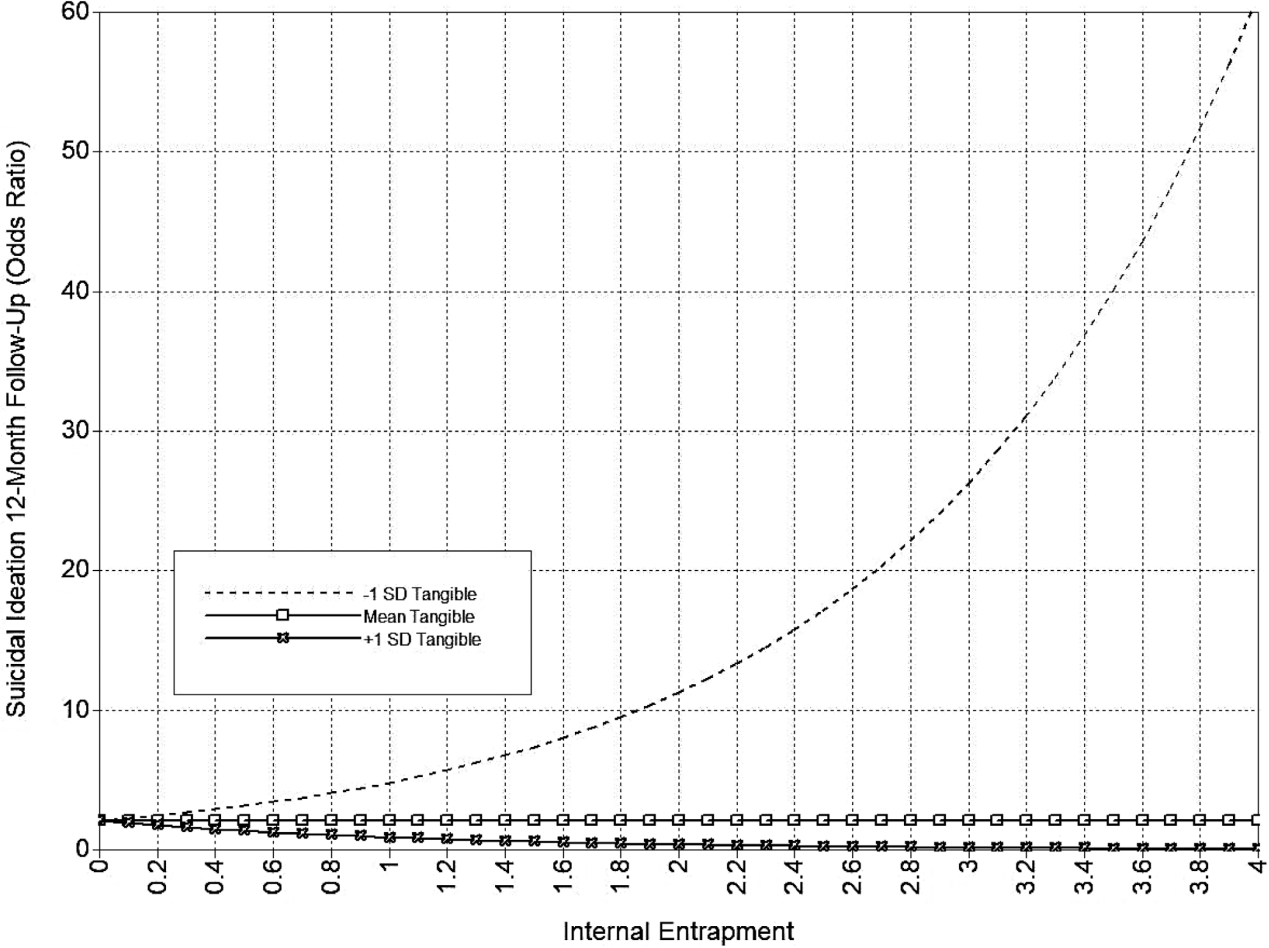
IE × tangible support interaction (SI 12‐month)—depression included as covariate. Predicted values from interaction of tangible support with Internal Entrapment in odds ratio scale of the outcome (suicidal ideation at 12‐month follow‐up). Internal Entrapment shown at logical values in the metric of the E‐Scale (0–4).

**FIGURE 9:**
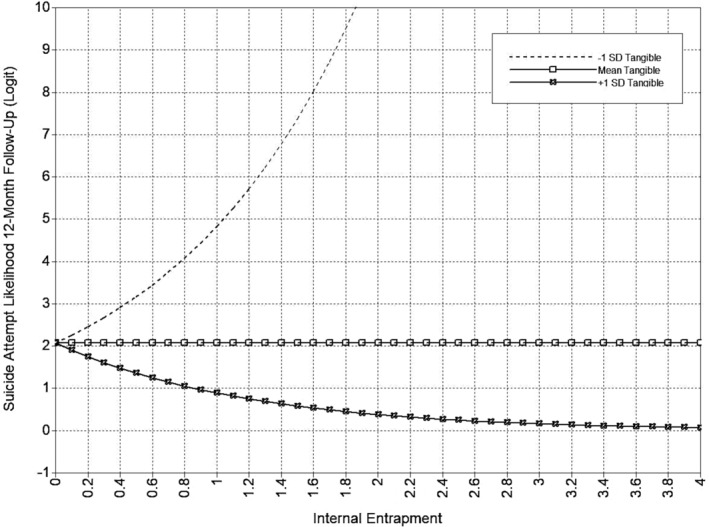
IE × Tangible support interaction (SA 12‐month)—depression included as covariate. Predicted values from interaction of tangible support with Internal Entrapment in logit scale of the outcome (suicide attempt likelihood at 12‐month follow‐up). Internal Entrapment shown at logical values in the metric of the E‐Scale (0–4).

The full tangible support model is presented in Table 5 and Figure 6. In the tangible support model (*LL* = −14,708.46; *AIC* = 29,590.92; *BIC* = 29,997.39), IE was significantly positively associated with both suicidal ideation and suicide attempt likelihood at 6‐month follow‐up. Neither EE nor any entrapment‐tangible support interaction terms were related to suicide outcomes at 6‐month follow‐up. EE was significantly positively associated with suicidal ideation at 12‐month follow‐up. Also, the interactions between EE and tangible support, as well as IE and tangible support, were significantly associated with suicidal ideation at 12‐month follow‐up. The EE‐suicidal ideation relationship was significant at low and high levels of tangible support, and this relationship strengthened at higher levels of tangible support (see Table 6; Figure 7). The IE‐suicidal ideation relationship was only significant at low levels of tangible support, suggesting that higher levels of tangible support mitigated the effects of IE (see Table 6; Figure 8). IE was not associated with suicidal ideation or suicide attempt likelihood at 12‐month follow‐up. EE was significantly positively related to suicide attempt likelihood at 12‐month follow‐up. The interaction between IE and tangible support was significant for suicidal attempt likelihood at 12‐month follow‐up. The IE‐suicide attempt likelihood relationship was significant across all levels of tangible support, but the strength of the association decreased at higher levels of tangible support (see Table 6; Figure 9). The interaction of EE and tangible support was not significant for suicide attempt likelihood at 12‐month follow‐up.

#### Appraisal model without depression as a covariate

The following results focus on what changed when excluding depression as a covariate, see Table S7 in the online supplemental for complete model comparison differences. The appraisal support model, without depression as a covariate (*LL* = −14,917.45, *AIC* = 30,000.91, *BIC* = 30,388.68) is presented in Table S8 and Figure S10 in the online supplemental. Primarily, IE without depression as a covariate is positively associated with suicidal ideation and suicide attempt likelihood at the 6‐month follow‐up period. EE is positively associated with suicidal ideation at the 6‐month follow‐up period, and the interaction between IE and appraisal support is no longer significant for suicide attempt likelihood at 12‐month follow‐up. Simple slopes analyses of the interaction between EE and appraisal support for suicide attempt likelihood at 6‐month follow‐up are presented in Figure S11 online. Comparisons of the full appraisal support model and appraisal support model with depression excluded as a covariate indicate a significant decrement to omnibus fit in the model with depression excluded by *LL*, *AIC*, and *BIC* (*LR* χ^2^(4) = 214.26, *p* < 0.0001; Δ*AIC* = −420.54; *ΔBIC* = −402.34).

#### Belonging support model without depression as a covariate

The belonging support model, without depression as a covariate (*LL* = −14,924.86, *AIC* = 30,015.73, *BIC* = 30,403.51) is presented in Table S8 and Figure S12 in the online supplemental. EE was significantly positively associated with suicide attempt likelihood at 6‐month, when removing depression as a covariate. IE was associated with suicidal ideation and suicide attempt likelihood at 6‐month follow‐up. Simple slopes analyses of the interaction between EE and belonging support for suicide attempt likelihood at 6‐month follow‐up are presented in Figure S13 online. Comparisons of the full belonging support model and belonging support model with depression excluded as a covariate indicate a significant decrement to omnibus fit in the model with depression excluded by *LL*, *AIC*, and *BIC* (*LR χ* ^2^(4) = 215.02, *p* < 0.0001; Δ*AIC* = −422.05; *ΔBIC* = −403.36).

#### Tangible support model without depression as a covariate

The tangible support model without depression as a covariate (*LL* = −14,921.36, *AIC* = 30,008.73, *BIC* = 30,396.51) is presented in Table S8 and Figure S14 in the online supplemental. The interaction between IE and tangible support is significant on suicide attempt likelihood at the 6‐ and 12‐month follow‐up. In the interaction between IE and tangible support on suicide attempt likelihood at 6‐month follow‐up, the association between IE and suicide attempt likelihood strengthens as tangible support increases (see Figure S15 online). In the interaction between IE and tangible support on suicide attempt likelihood at 12‐month follow‐up, the association between IE and suicide attempt likelihood decreases as tangible support increases (see Table S9 online; Figure S16 online). The interaction effects between IE and EE with tangible support are no longer significant when depression is not included as a covariate. Trauma alone was not associated with either suicidal ideation or suicide attempt likelihood at any of the follow‐up periods. Comparisons of the full tangible support model and tangible support model with depression excluded as a covariate indicate a significant decrement to omnibus fit in the model with depression excluded by *LL*, *AIC*, and *BIC* (*LR χ* ^2^(4) = 212.90, *p* < 0.0001; Δ*AIC* = −417.81; *ΔBIC* = −399.12).

## DISCUSSION

In this study, we investigated the factor structure, reliability, and predictive validity of the E‐Scale among active‐duty service members, dependents of active‐duty service members, and military retirees recruited from five separate US military installations. E‐Scale factor structures indicated superior data fit for the two‐factor solution over a single‐factor solution, consistent with prior research (Cramer et al., 2019; Forkmann et al., 2018). IE and EE were also found to be highly correlated, as seen in previous research (Gilbert & Allan, 1998), indicating that even though IE and EE are distinct constructs, they do have shared variance. Although some have argued for a single‐factor solution for entrapment (Griffiths et al., 2014; Taylor et al., 2009), most recent efforts have supported our findings that IE and EE are related but distinct constructs (Cramer et al., 2019; Höller et al., 2022; Owen et al., 2018).

We next evaluated associations of EE and IE with later suicidal ideation and suicide attempt likelihood. Our findings correspond with the preponderance of evidence supporting the positive association between entrapment and suicidal ideation (O'Connor & Portzky, 2018; Siddaway et al., 2015). However, as we distinguish between the two factors within entrapment, a more complex picture emerges. Our results suggest that both IE and EE were predictive of suicidal ideation and suicide attempt likelihood, with effects differing somewhat across models. Specifically, EE was directly and positively associated with suicide attempt likelihood at 6‐month follow‐up (appraisal only) and both suicidal ideation and suicide attempt likelihood at 12‐month follow‐up (all social support models). IE was directly and positively associated with suicidal ideation and suicide attempt likelihood at 6‐month follow‐up (tangible support only).

Our observed results showing both IE (at 6‐month) and EE (at 6‐ and 12‐ month) predict suicidal ideation and suicide attempt likelihood at follow‐up time‐points diverges somewhat from prior research that found support for IE as the stronger predictive factor, especially overtime (Höller et al., 2022; Owen et al., 2018; Rasmussen et al., 2010). For instance, Höller et al. (2022) found that IE, but not EE, predicted suicidal ideation over a 12‐month time frame in a sample of post‐discharge psychiatric patients. Our findings regarding EE contradict Höller et al.'s results. The direct impacts of IE and EE on suicide outcomes over time appears to be context dependent. For psychiatric patients, the nature of IE (e.g., overlap with depression; feeling trapped in one's own thoughts) may explain why it is a more prominent suicidal ideation risk factor. While our findings also show IE may be linked with suicidal ideation in the short‐term, EE may reflect the more long‐standing social challenges endured by military service members, retirees, and their families (e.g., separation, changes in duty assignments). In other words, EE may be a unique risk factor for suicidal ideation and suicide attempt likelihood due to the social context of military life.

Speaking to the contextual influence of military culture on EE and suicide, there is increased importance of EE and its impact on suicide risk in a military‐affiliated sample compared to prior research with civilians. EE may be both a common and unique experience for service members, retirees, and their families. For instance, active‐duty service members and their families share much in common with respect to social/interpersonal challenges that may be experienced as EE (e.g., reintegration to civilian society, deployment, access to support). Unique aspects of EE for service members may reflect relinquishing control over multiple domains—frequent duty station changes, high operational deployment cycles, lack of autonomy within job role and the command structure—that have potentially deleterious effects on psychological health (Brooks & Greenberg, 2018; Thomas et al., 2005). For military family members, EE may also entail career and social disruptions due to frequent relocations or living in rural or austere environments with limited employment options (Gonzalez et al., 2015). Thus, military service members and their families may be more likely to experience EE and subsequently have it contribute to suicidal ideation in contrast to civilian samples.

We further examined the moderating effects of social support on the association between entrapment and suicidal ideation. While the overall picture is that social support does play a role in the pathway to suicidal ideation and suicide attempt likelihood, the story was complex. Consistent with prior hypotheses, there was a significant interaction between tangible support and IE on suicidal ideation and suicide attempt likelihood at 12‐months follow‐up. IE was positively associated with suicidal ideation and suicide attempt likelihood at low levels of tangible support; however, the association between IE was attenuated and at higher levels of tangible support. Tangible social support appears to buffer the negative impacts of IE on suicidal ideation and suicide attempt likelihood, which is consistent with other research supporting a positive buffering effect of social support on suicide risk (Panagioti et al., 2014). Previous research with active‐duty military personnel has demonstrated that the top reported reasons for attempting suicide include reducing uncomfortable and painful internal psychological distress (Bryan et al., 2013). Having someone to turn to for help (tangible support) may help individuals solve or resolve their internal pain or sense of feeling trapped in their pain (IE).

When examining other forms of social support, several significant interactions were unexpected. Higher levels of appraisal support strengthened the association between EE and suicide attempt likelihood at the 6‐month follow‐up. Similar associations were found for IE and appraisal support for suicide attempt likelihood at 12‐month follow‐up, for EE and belonging support for suicide attempt likelihood at 6‐month follow‐up, and for EE and tangible support for suicidal ideation at 12‐month follow‐up. In each of these cases, the relationship between entrapment and STBs was not diminished but rather enhanced in the presence of high social support. Social support takes time to develop in interpersonal relationships and may not be as accessible to members of the military who experience frequent job relocations (Borah & Fina, 2017). This may be particularly true for those who endorse strong social support, but due to factors related to the military are unable to adequately access their support systems during times of crisis. When crises emerge, they may feel increasingly stuck and alone, amplifying the relationship between entrapment and STBs. Additionally, relying on social support alone is likely insufficient to counter systemic concerns highlighted in the recent SPRIRC report that noted several environmental factors potentially heightening suicide in the military (SPRIRC, 2023).

We also ran additional models, with depression excluded as a covariate for each type of social support (i.e., appraisal, belonging, and tangible). These additional analyses were conducted due to the relationship among the arrested flight model, entrapment, and depression (e.g., Gilbert & Allan, 1998). Relationships largely remained the same with a few key differences; while a few significant effects dropped out, we focus on what additions emerged in new models. Notably, within the appraisal support model both EE and IE were positively associated with suicidal ideation at the 6‐month follow‐up period and IE was also now positively associated with suicide attempt likelihood at the 6‐month follow‐up period. Similarly, within the belonging support model IE was positively associated with suicidal ideation and suicide attempt likelihood at the 6‐month follow‐up period. Emergence of new significant IE and EE effects suggest that depression may suppress the entrapment‐suicide link, particularly for IE on suicidal ideation and suicide attempt likelihood.

Within the tangible support models, a new significant two‐way interaction effect emerged for IE and tangible support on suicide attempt likelihood at 6‐ and 12‐month follow‐up. The pattern of this effect was contrary to expected stress buffering patterns for social support. The IE‐suicide attempt likelihood link was positive at high levels of tangible support and vice versa; tangible support exacerbated the IE impact of suicide attempt likelihood at 6‐months (see Online Figure S15). The IE by tangible support interaction reverts to expected patterns in the long‐term; at 12‐months, IE is positively predictive of suicide attempt likelihood, suggesting a stress‐buffering protective role (see Online Figure S16). The Military Family Well‐being and Readiness Model (Bowles et al., 2015; NASEM, 2019) holds that social facets of military life (e.g., transition into the military, deployment, reintegration to civilian life) can prove to be both challenges and opportunities (Bowles et al., 2015). Tangible supports at the familial (e.g., coping skills) and military (e.g., military community resources) level play a pivotal role whether such circumstances negatively or positively impact well‐being. In the instance of suicide attempt likelihood, having tangible social support may create more short‐term strain than actual benefit when faced with social challenges in the military (e.g., having more family members to account for logistically [during a move], emotionally or financially). Additionally, service members and their families initially having tangible support networks may not equate to knowing how to utilize such a resource. As a result, near term (i.e., within 6‐months) strains and confusion posed by tangible social support in a military context may worsen feelings of being trapped and seeing suicide as an escape. Over time and with adjustment, military service members and their families may adapt to military social challenges and learn how to leverage supports in the military system, resulting in a protective role of tangible support in the long‐term (12‐month follow‐up).

Overall, our findings have implications for the IMV (O'Connor, 2011; O'Connor & Kirtley, 2018), and its need for possible refinement. First, our findings suggest the IMV hypothesized direct path to suicidal ideation and eventually suicide attempt may be more nuanced. In conjunction with other literature (e.g., Höller et al., 2022), the IMV may need to be revised by having parallel pathways from suicidal ideation, one IE and one EE. The EE should further be demarcated in some manner as context‐dependent based on our and other findings. Second, the IMV also posits social support should be a motivational phase protective factor. Our observed moderating effects of social support are not that straightforward. All instances of significant social support moderation effects for appraisal and belonging support yielded entrapment exacerbation, contrary to the generally protective role of social support in the broader health literature (e.g., Cohen et al., 1985; Helgeson, 2003). On the other hand, tangible support served the predicted buffering role at 12‐months, reducing the impact of IE on suicidal ideation and suicide attempt likelihood over a relatively long time‐period. Thus, tangible social support appears to have protective value when military‐affiliated persons feel trapped by their own thoughts. Therefore, we suggest that the motivational moderator aspect of the IMV may need to be considered context dependent. In terms of future IMV model exploration, a more fine‐grained consideration of the potential risk‐buffering or risk‐amplifying impacts of different types of social support would be beneficial. Finally, presence of depression in models has clear impact on findings, and therefore possible interpretation of the IMV. Future IMV research should carefully consider the role of depression in ways such as collinearity with IE and EE, or a pre‐motivational driver of IMV pathways, especially for samples at high risk for depression.

The study was not without limitations. First, there were limited measurement of suicide outcomes. Ideation was binary, and relied on retrospective self‐report and we know measuring suicidal ideation is far more complex (Wastler et al., 2023). Future data collection methods should implement daily diary or ecological momentary assessment methods to improve reporting accuracy of current suicidal ideation. Our metric of intent was imprecise, with intent inconsistently measured in the literature (Freedenthal, 2007). Future research should examine IMV pathways using dimensional metrics of STBs. Second, social support measure reliability was low. Low reliability may have restricted discovery of other significant associations or moderating effects (Nimon et al., 2012). Future research may employ other established social support measures (e.g., Multidimensional Social Support measure; Zimet et al., 1988) or other motivational moderators (e.g., test sense of belonging) to gain insight into the (in)consistency of our social support findings. Additionally, only those eligible to receive care within a military healthcare system, to include active‐duty service members, military retirees, and their families, were included in this study and results may not generalize outside of this population. However, inclusion of military retirees and family members who often share social/interpersonal challenges with active‐duty service members (e.g., reintegration to civilian society, deployment, access to support; for example, Ormeno et al., 2020; Strong & Lee, 2017) is an overall strength of the study and may help to inform joint efforts to reduce shared risk within the military community (e.g., SAMHSA, 2023). Overall, findings from this study support a two‐factor structure of entrapment within a military context. EE appears most salient in increasing suicide risk for those in the military, with tangible support offering potential mitigative effects in reducing overall suicide risk.

## CONFLICT OF INTEREST STATEMENT

The authors report no conflict of interest.

## Supporting information


Data S1.

## Data Availability

The data that support the findings of this study are available from the corresponding author upon reasonable request.

## Endnotes

^i^
Item examples from the E‐Scale for external and internal entrapment are provided in the measures section.

^ii^
Each autoregressive model yielded evidence of model instability. Additionally, suicidal ideation and attempt likelihood yielded poor covariance coverage (ranging from 0.480 to 0.562) with both internal and external entrapment items, suggesting localized ill‐fit within each model.
