# Supplementary material for: Entrapment in the military context: Factor structure and associations with suicidal thoughts and behaviors
Source: Suicide Life Threat Behav. 2024 Aug 13;54(6):1006–28. doi: 10.1111/sltb.13105 (PMC11629605; doi:10.1111/sltb.13105)
Supplement: Supplementary file 1 — Data S1. [file SLTB-54-1006-s001.docx]

**Supplemental - DEPRESSION EXCLUDED**

Table 7. Comparison of Parameter Estimate Changes between Models with and without Depression as a Covariate

| Model | Predictor | 6 mo. SI (Full) | 6 mo. SI (Dep. Excl) | 6 mo. SA (Full) | 6 mo. SA (Dep Excl) | 12 mo. SI (Full) | 12 mo. SI (Dep Excl) | 12 mo. SA (Full) | 12 mo. SA (Dep Excl) |
| --- | --- | --- | --- | --- | --- | --- | --- | --- | --- |
| Appraisal Support | | | | | | | | | |
|  | EE | ***ns*** | **sig** | sig | sig | sig | sig | sig | sig |
|  | IE | ***ns*** | **sig** | ***ns*** | **sig** | *ns* | *ns* | *ns* | *ns* |
|  | EE x Support | *ns* | *ns* | sig | sig | *ns* | *ns* | *ns* | *ns* |
|  | IE x Support | *ns* | *ns* | *ns* | *ns* | *ns* | *ns* | **sig** | ***ns*** |
|  | Trauma Exposure | *ns* | *ns* | *ns* | *ns* | *ns* | *ns* | *ns* | *ns* |
|  | Depression | sig | *** | sig | *** | *ns* | *** | *ns* | *** |
|  | Support | *ns* | *ns* | *ns* | *ns* | *ns* | *ns* | *ns* | *ns* |
| Belonging Support | | | | | | | | | |
|  | EE | *ns* | *ns* | ***ns*** | **sig** | sig | sig | sig | sig |
|  | IE | ***ns*** | **sig** | ***ns*** | **sig** | *ns* | *ns* | *ns* | *ns* |
|  | EE x Support | *ns* | *ns* | sig | sig | *ns* | *ns* | *ns* | *ns* |
|  | IE x Support | *ns* | *ns* | *ns* | *ns* | *ns* | *ns* | *ns* | *ns* |
|  | Trauma Exposure | *ns* | *ns* | *ns* | *ns* | *ns* | *ns* | *ns* | *ns* |
|  | Depression | sig | *** | sig | *** | *ns* | *** | *ns* | *** |
|  | Support | *ns* | *ns* | *ns* | *ns* | sig | sig | ***ns*** | **sig** |
| Tangible Support | | | | | | | | | |
|  | EE | *ns* | *ns* | *ns* | *ns* | sig | sig | sig | sig |
|  | IE | sig | sig | sig | sig | *ns* | *ns* | *ns* | *ns* |
|  | EE x Support | *ns* | *ns* | *ns* | *ns* | **sig** | ***ns*** | *ns* | *ns* |
|  | IE x Support | *ns* | *ns* | ***ns*** | **sig** | **sig** | ***ns*** | sig | sig |
|  | Trauma Exposure | *ns* | *ns* | *ns* | *ns* | *ns* | *ns* | *ns* | *ns* |
|  | Depression | sig | *** | sig | *** | *ns* | *** | *ns* | *** |
|  | Support | **sig** | ***ns*** | *ns* | *ns* | sig | sig | *ns* | *ns* |
| Note: Bolded terms highlight changes when depression was removed as a covariate | | | | | |  |  |  |  |

Table 8. Entrapment and Support Type Autoregressive Model Statics - Depression Excluded as Covariate

| Outcome | Predictor | Coef. | *S.E.* | *OR* | *p* | 95% CI | Coef. | *S.E.* | *OR* | *p* | 95% CI | Coef. | *S.E.* | *OR* | *p* | 95% CI |
| --- | --- | --- | --- | --- | --- | --- | --- | --- | --- | --- | --- | --- | --- | --- | --- | --- |
|  |  | Appraisal Support | | | | | Belonging Support | | | | | Tangible Support | | | | |
| 6 mo. SI | EE | **0.57** | **0.26** | **1.77** | **.031** | **1.06 \| 2.97** | 0.15 | 0.30 | 1.16 | .620 | 0.65 \| 2.08 | 0.07 | 0.30 | 1.07 | .822 | 0.60 \| 1.91 |
|  | IE | **0.66** | **0.24** | **1.94** | **.007** | **1.20 \| 3.12** | **0.91** | **0.28** | **2.48** | **.001** | **1.43 \| 4.30** | **1.10** | **0.29** | **3.01** | **< .001** | **1.71 \| 5.28** |
|  | EE x Support | 0.04 | 0.08 | 1.04 | .624 | 0.90 \| 1.20 | 0.10 | 0.09 | 1.10 | .263 | 0.93 \| 1.31 | -0.06 | 0.08 | 0.94 | .427 | 0.80 \| 1.10 |
|  | IE x Support | -0.02 | 0.07 | 0.98 | .819 | 0.86 \| 1.13 | -0.06 | 0.08 | 0.95 | .506 | 0.80 \| 1.11 | 0.10 | 0.07 | 1.11 | .150 | 0.96 \| 1.28 |
|  | Trauma Exposure | -0.01 | 0.05 | 0.99 | .863 | 0.90 \| 1.09 | -0.04 | 0.05 | 0.96 | .437 | 0.67 \| 1.06 | -0.05 | 0.05 | 0.95 | .323 | 0.86 \| 1.05 |
|  | Support | 0.06 | 0.06 | 1.07 | .314 | 0.94 \| 1.20 | 0.01 | 0.05 | 1.01 | .794 | 0.91 \| 1.13 | 0.09 | 0.06 | 1.09 | .150 | 0.97 \| 1.24 |
| 6 mo. SA | EE | **0.35** | **0.10** | *** | **< .001** | *** | **0.21** | **0.11** | ******* | **.045** | ******* | 0.19 | 0.10 | *** | .070 | *** |
|  | IE | **0.18** | **0.09** | ******* | **.042** | ******* | **0.38** | **0.10** | ******* | **< .001** | ******* | **0.43** | **0.10** | *** | **< .001** | *** |
|  | EE x Support | **0.05** | **0.03** | *** | **.034** | *** | **0.06** | **0.03** | *** | **.043** | *** | -0.04 | 0.03 | *** | .180 | *** |
|  | IE x Support | -0.03 | 0.02 | *** | .137 | *** | -0.03 | 0.03 | *** | .279 | *** | **0.08** | **0.03** | ******* | **.003** | *** |
|  | Trauma Exposure | -0.01 | 0.02 | *** | .393 | *** | -0.01 | 0.02 | *** | .726 | *** | 0.00 | 0.02 | *** | .818 | *** |
|  | Support | 0.02 | 0.02 | *** | .220 | *** | 0.01 | 0.02 | *** | .486 | *** | 0.02 | 0.02 | *** | .317 | *** |
| 12 mo. SI | EE | **0.98** | **0.38** | **2.66** | **.009** | **1.28 \| 5.54** | **1.21** | **0.38** | **3.35** | **.002** | **1.58 \| 7.09** | **1.29** | **0.39** | **3.64** | **.001** | **1.70 \| 7.82** |
|  | IE | 0.00 | 0.37 | 1.00 | .999 | 0.49 \| 2.04 | -0.31 | 0.37 | 0.74 | .409 | 0.36 \| 1.52 | -0.46 | 0.37 | 0.63 | .223 | 0.30 \| 1.32 |
|  | EE x Support | -0.01 | 0.11 | 0.99 | .924 | 0.81 \| 1.22 | 0.05 | 0.10 | 1.05 | .623 | 0.86 \| 1.28 | 0.29 | 0.15 | 1.34 | .058 | 0.99 \| 1.81 |
|  | IE x Support | 0.14 | 0.10 | 1.11 | .285 | 0.92 \| 1.34 | 0.01 | 0.09 | 1.01 | .882 | 0.84 \| 1.22 | -0.22 | 0.14 | 0.80 | .098 | 0.61 \| 1.04 |
|  | Trauma Exposure | -0.14 | 0.08 | 0.87 | .070 | 0.74 \| 1.01 | -0.12 | 0.08 | 0.89 | .128 | 0.76 \| 1.04 | -0.12 | 0.08 | 0.89 | .147 | 0.76 \| 1.04 |
|  | Support | -0.06 | 0.07 | 0.94 | .384 | 0.82 \| 1.08 | **-0.15** | **0.06** | **0.86** | **.015** | **0.76 \| 0.97** | **-0.18** | **0.08** | **0.83** | **.014** | **0.72 \| 0.96** |
|  | 6 mo. SI | **2.29** | **0.53** | **9.87** | **< .001** | **3.51 \| 27.70** | **2.40** | **0.51** | **11.05** | **< .001** | **4.10 \| 29.78** | **2.54** | **0.52** | **12.66** | **< .001** | **4.58 \| 35.03** |
|  | 6 mo. SA | 0.09 | 0.23 | 1.09 | .708 | 0.70 \| 1.70 | 0.10 | 0.22 | 1.10 | .653 | 0.72 \| 1.68 | 0.12 | 0.22 | 1.12 | .602 | 0.73 \| 1.74 |
| 12 mo. SA | EE | **0.22** | **0.08** | *** | **.004** | *** | **0.16** | **0.08** | *** | **.034** | *** | **0.16** | **0.08** | *** | **.036** | *** |
|  | IE | -0.03 | 0.08 | *** | .628 | *** | -0.02 | 0.08 | *** | .775 | *** | -0.04 | 0.08 | *** | .628 | *** |
|  | EE x Support | -0.03 | 0.03 | *** | .271 | *** | -0.05 | 0.03 | *** | .070 | *** | 0.03 | 0.02 | *** | .269 | *** |
|  | IE x Support | 0.05 | 0.02 | *** | .060 | *** | 0.04 | 0.02 | *** | .142 | *** | **-0.05** | **0.02** | *** | **.027** | *** |
|  | Trauma Exposure | 0.00 | 0.01 | *** | .988 | *** | 0.00 | 0.01 | *** | .798 | *** | 0.00 | 0.01 | *** | .857 | *** |
|  | Support | 0.00 | 0.01 | *** | .956 | *** | **-0.03** | **0.01** | *** | **.021** | *** | -0.02 | 0.01 | *** | .098 | *** |
|  | 6 mo. SI | **0.24** | **0.05** | *** | **< .001** | *** | **0.25** | **0.05** | *** | **< .001** | *** | **0.25** | **0.05** | *** | **< .001** | *** |
|  | 6 mo. SA | **1.21** | **0.12** | *** | **< .001** | *** | **1.21** | **0.12** | ******* | **< .001** | *** | **1.22** | **0.12** | ******* | **< .001** | *** |

Note: S. E. = Standard error; OR = Odds ratio; CI = Confidence interval; 6 mo. SI = Suicidal ideation at 6-month follow-up; 6 mo. SA = Suicide attempt likelihood at 6-month follow-up; 12 mo. SI = Suicidal ideation at 12-month follow-up; 12 mo. SA = Suicide attempt likelihood at 12-month follow-up; IE = Internal entrapment; EE = External entrapment; Support = Support type (Appraisal, Belonging, Tangible); x = Interaction term. SA model outcomes are continuous while SI model outcomes are binary. Bolded terms indicate significance.

Table 9. Autoregressive Model Social Support Simple Slopes Analyses - Depression Excluded as Covariate

| Outcome | Interaction | Level | Coef. | *p* | 95%CI |
| --- | --- | --- | --- | --- | --- |
|  |  |  |  |  |  |
| 6 mo. SA | EE x App |  |  |  |  |
|  |  | -1*SD* | 0.912 | < .05 | 0.65 \| 1.18 |
|  |  | Mean | 1.077 | < .05 | 0.84 \| 1.31 |
|  |  | +1*SD* | 1.242 | < .05 | 0.95 \| 1.53 |
|  | EE x Belong | |  |  |  |
|  |  | -1*SD* | 0.970 | < .05 | 0.68 \| 1.26 |
|  |  | Mean | 1.148 | < .05 | 0.91 \| 1.39 |
|  |  | +1*SD* | 1.325 | < .05 | 1.03 \| 1.63 |
|  | IE x Tang |  |  |  |  |
|  |  | -1*SD* | 0.946 | < .05 | 0.65 \| 1.24 |
|  |  | Mean | 1.172 | < .05 | 0.92 \| 1.42 |
|  |  | +1*SD* | 1.399 | < .05 | 1.11 \| 1.69 |
| 12 mo. SA | IE x Tang |  |  |  |  |
|  |  | -1*SD* | 1.908 | < .05 | 1.63 \| 2.19 |
|  |  | Mean | 1.760 | < .05 | 1.51 \| 2.02 |
|  |  | +1*SD* | 1.612 | < .05 | 1.32 \| 1.91 |

Note: +/- 1 SD = One standard deviation above or below the mean; OR = Odds ratio; p = *p*-value; CI = Confidence interval; 6 mo. SI = Suicidal ideation at 6 month follow-up; 6 mo. SA = Suicide attempt likelihood at 6 month follow-up; 12 mo. SI = Suicidal ideation at 12 month follow-up; 12 mo. SA = Suicide attempt likelihood at 12 month follow-up; IE = Internal entrapment; EE = External entrapment; App = Appraisal support; Belong = Belonging support; Tang = Tangible support; x = Interaction term.

**Figure 10**

*Appraisal Support Autoregressive Model - Depression Excluded as Covariate*

*
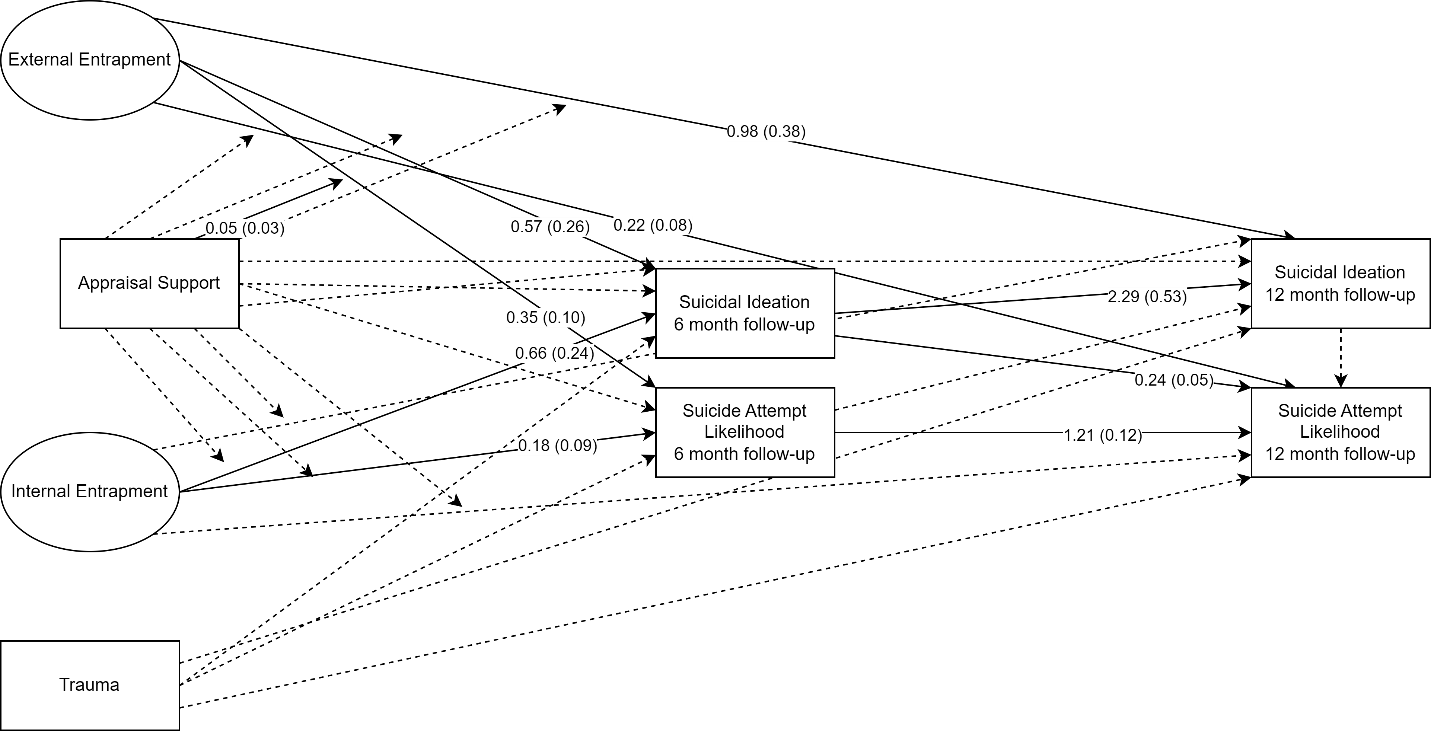
*

*Note.* Autoregressive structural equation model results shown with circles representing latent variables (External and Internal entrapment) and rectangles indicating observed variables. Parameter estimates for significant paths shown as unstandardized logistic regression coefficients with standard errors in parentheses. Solid lines indicate significant paths. Dashed lines indicate nonsignificant paths. Arrows pointing directly to variables indicate direct associations between variables, while arrows pointing to paths indicate interactions. Variances and disturbances not shown for brevity.

**Figure 11**

*EE x Appraisal Support Interaction (SA 6-month) - Depression Excluded as Covariate*


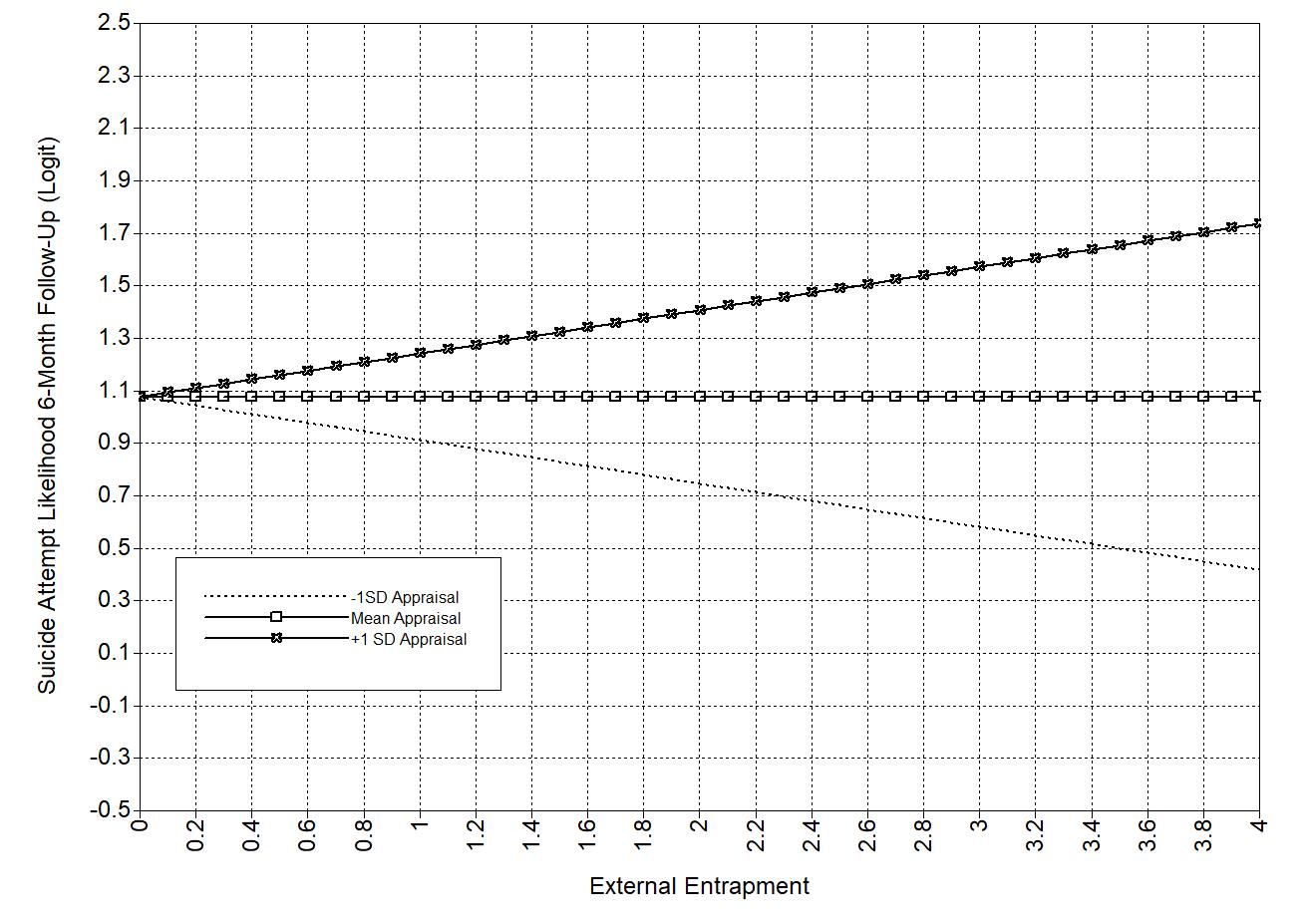


*Note*. Predicted values from interaction of appraisal support with External Entrapment in logit scale of the outcome (suicide attempt likelihood at 6-month follow-up). External Entrapment shown at logical values in the metric of the E-Scale (0 to 4).

**Figure 12**

*Belonging Support Autoregressive Model - Depression Excluded as Covariate*


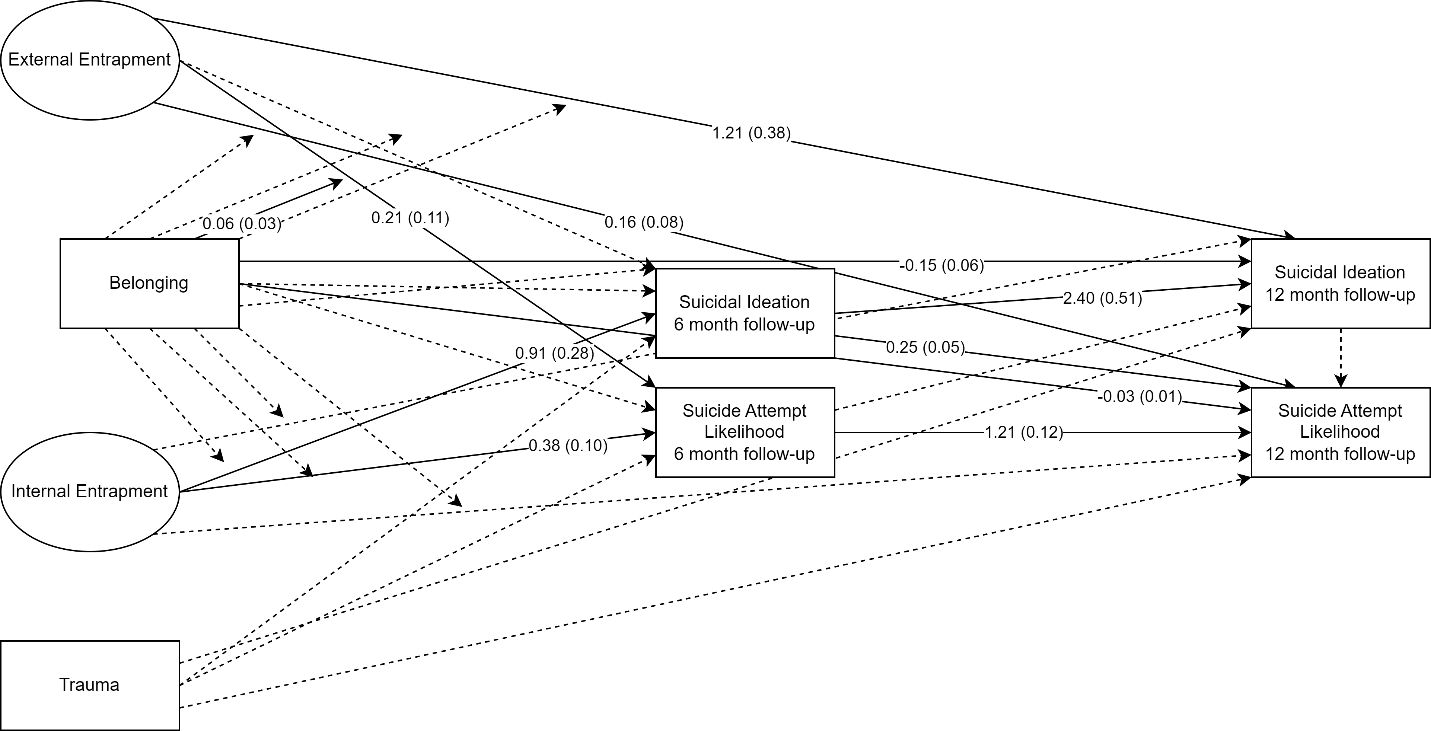


*Note.* Autoregressive structural equation model results shown with circles representing latent variables (External and Internal entrapment) and rectangles indicating observed variables. Parameter estimates for significant paths shown as unstandardized logistic regression coefficients with standard errors in parentheses. Solid lines indicate significant paths. Dashed lines indicate nonsignificant paths. Arrows pointing directly to variables indicate direct associations between variables, while arrows pointing to paths indicate interactions. Variances and disturbances not shown for brevity.

**Figure 13**

*EE x Belonging Support Interaction (SA 6-month) - Depression Excluded as Covariate*


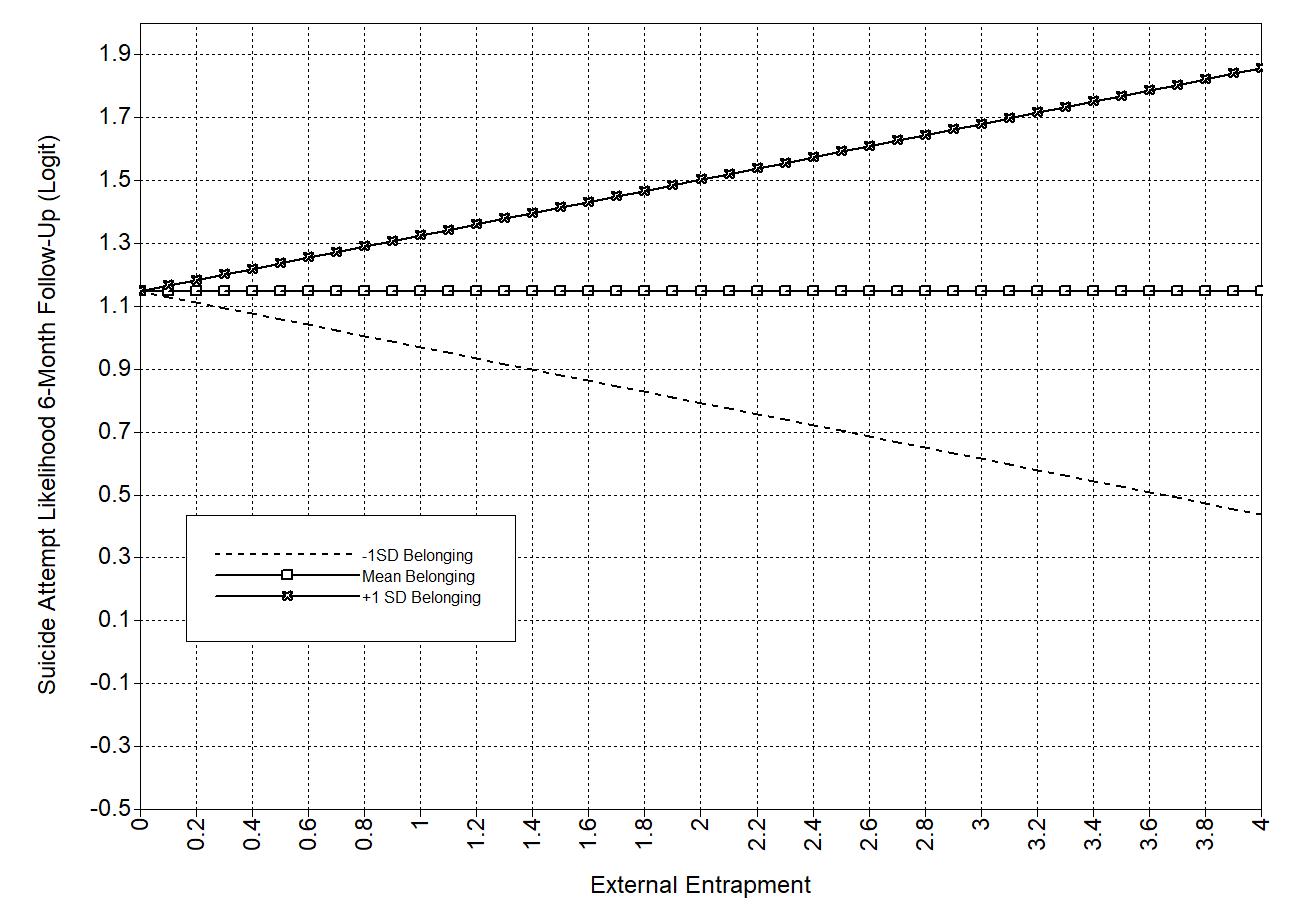


*Note*. Predicted values from interaction of belonging support with External Entrapment in logit scale of the outcome (suicide attempt likelihood at 6-month follow-up). External Entrapment shown at logical values in the metric of the E-Scale (0 to 4).

**Figure 14**

*Tangible Support Autoregressive Model - Depression Excluded as Covariate*


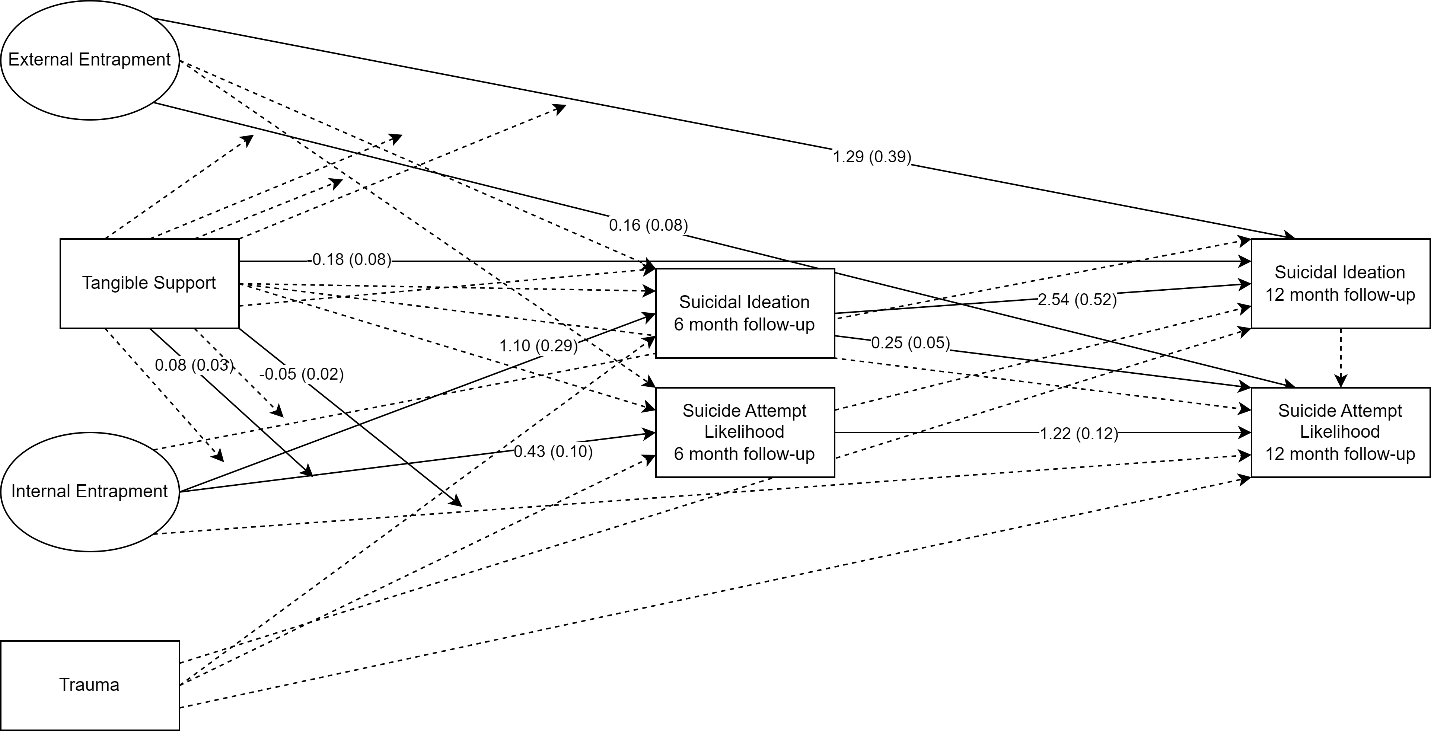


*Note.* Autoregressive structural equation model results shown with circles representing latent variables (External and Internal entrapment) and rectangles indicating observed variables. Parameter estimates for significant paths shown as unstandardized logistic regression coefficients with standard errors in parentheses. Solid lines indicate significant paths. Dashed lines indicate nonsignificant paths. Arrows pointing directly to variables indicate direct associations between variables, while arrows pointing to paths indicate interactions. Variances and disturbances not shown for brevity.

**Figure 15**

*IE x Tangible Interaction (SA 6-month) - Depression Excluded as Covariate*

**
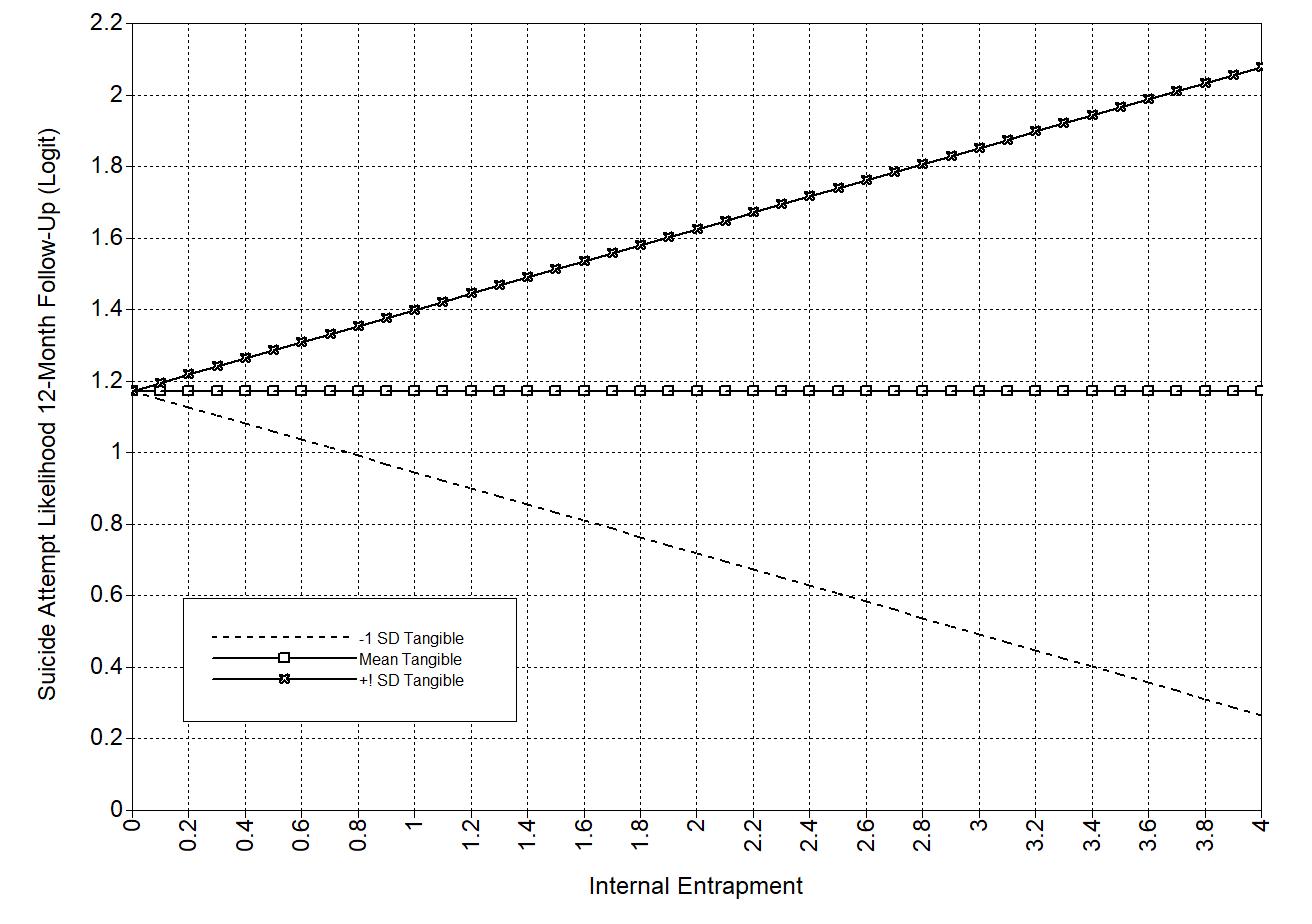
**

*Note*. Predicted values from interaction of tangible support with Internal Entrapment in logit scale of the outcome (suicide attempt likelihood at 6-month follow-up). Internal Entrapment shown at logical values in the metric of the E-Scale (0 to 4).

**Figure 16**

*IE x Tangible Interaction (SA 12-month) - Depression Excluded as Covariate*


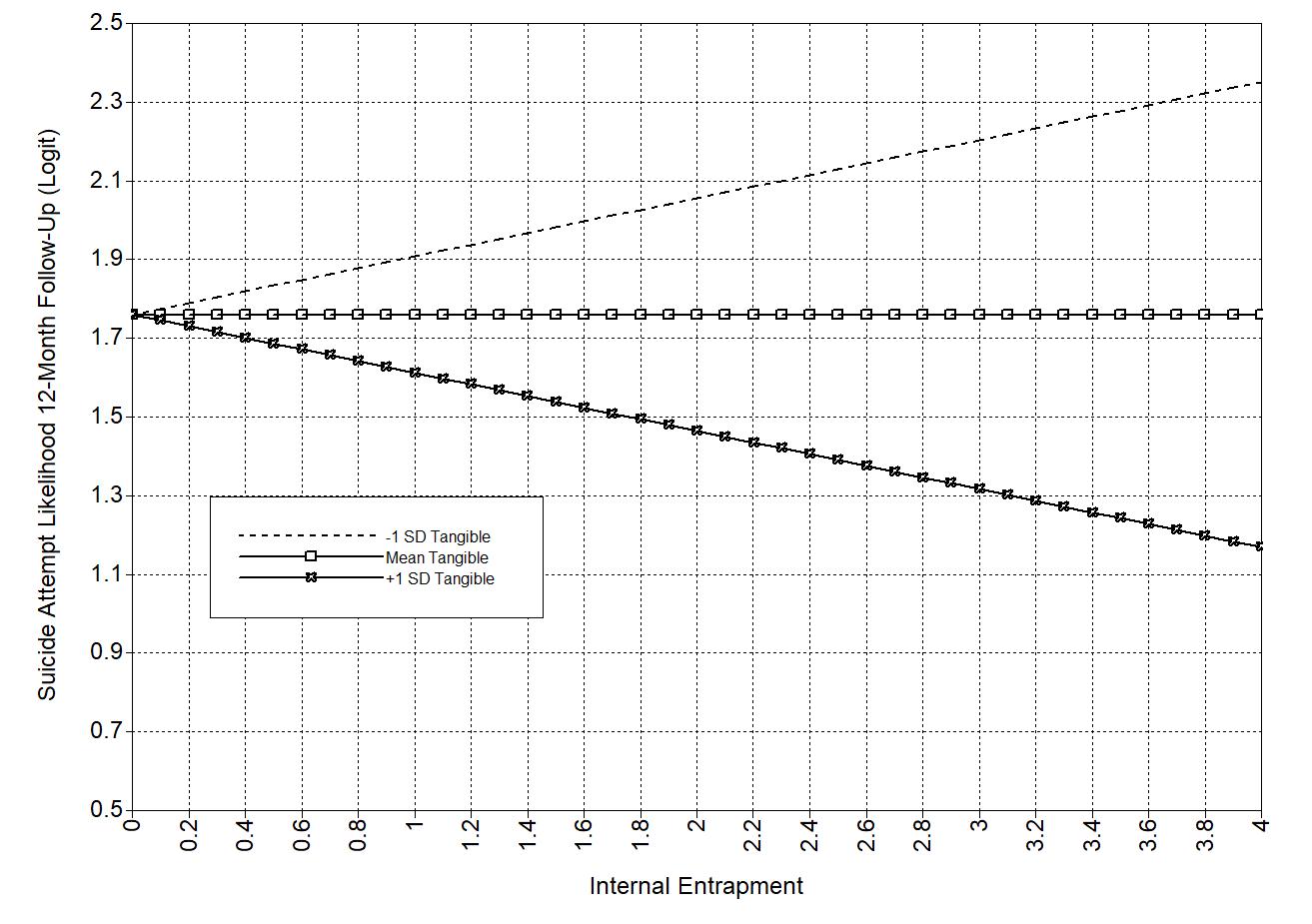


*Note*. Predicted values from interaction of tangible support with Internal Entrapment in logit scale of the outcome (suicide attempt likelihood at 12-month follow-up). Internal Entrapment shown at logical values in the metric of the E-Scale (0 to 4).
